# Biochemical basis for the formation of organ-specific volatile blends in mint

**DOI:** 10.3389/fpls.2023.1125065

**Published:** 2023-04-14

**Authors:** B. Markus Lange, Narayanan Srividya, Iris Lange, Amber N. Parrish, Lukas R. Benzenberg, Iovanna Pandelova, Kelly J. Vining, Matthias Wüst

**Affiliations:** ^1^ Institute of Biological Chemistry and M.J. Murdock Metabolomics Laboratory, WashingtonState University, Pullman, WA, United States; ^2^ Institut für Ernährungs- und Lebensmittelwissenschaften, Rheinische Friedrich Wilhelms-UniversitätBonn, Bonn, Germany; ^3^ Department of Horticulture, Oregon State University, Corvallis, OR, United States

**Keywords:** Mentha, mint, monoterpene synthase, terpene, Verticillium, volatile

## Abstract

Above-ground material of members of the mint family is commercially distilled to extract essential oils, which are then formulated into a myriad of consumer products. Most of the research aimed at characterizing the processes involved in the formation of terpenoid oil constituents has focused on leaves. We now demonstrate, by investigating three mint species, peppermint (*Mentha* ˣ *piperita* L.), spearmint (*Mentha spicata* L.) and horsemint (*Mentha longifolia* (L.) Huds.; accessions CMEN 585 and CMEN 584), that other organs – namely stems, rhizomes and roots – also emit volatiles and that the terpenoid volatile composition of these organs can vary substantially from that of leaves, supporting the notion that substantial, currently underappreciated, chemical diversity exists. Differences in volatile quantities released by plants whose roots had been dipped in a *Verticillium dahliae*-spore suspension (experimental) or dipped in water (controls) were evident: increases of some volatiles in the root headspace of mint species that are susceptible to Verticillium wilt disease (peppermint and *M. longifolia* CMEN 584) were detected, while the quantities of certain volatiles decreased in rhizomes of species that show resistance to the disease (spearmint and M. longifolia CMEN 585). To address the genetic and biochemical basis underlying chemical diversity, we took advantage of the newly sequenced *M. longifolia* CMEN 585 genome to identify candidate genes putatively coding for monoterpene synthases (MTSs), the enzymes that catalyze the first committed step in the biosynthesis of monoterpenoid volatiles. The functions of these genes were established by heterologous expression in *Escherichia coli*, purification of the corresponding recombinant proteins, and enzyme assays, thereby establishing the existence of MTSs with activities to convert a common substrate, geranyl diphosphate, to (+)-α-terpineol, 1,8-cineole, γ-terpinene, and (–)-bornyl diphosphate, but were not active with other potential substrates. In conjunction with previously described MTSs that catalyze the formation of (–)-β-pinene and (–)-limonene, the product profiles of the MTSs identified here can explain the generation of all major monoterpene skeletons represented in the volatiles released by different mint organs.

## Introduction

**Figure 1:**
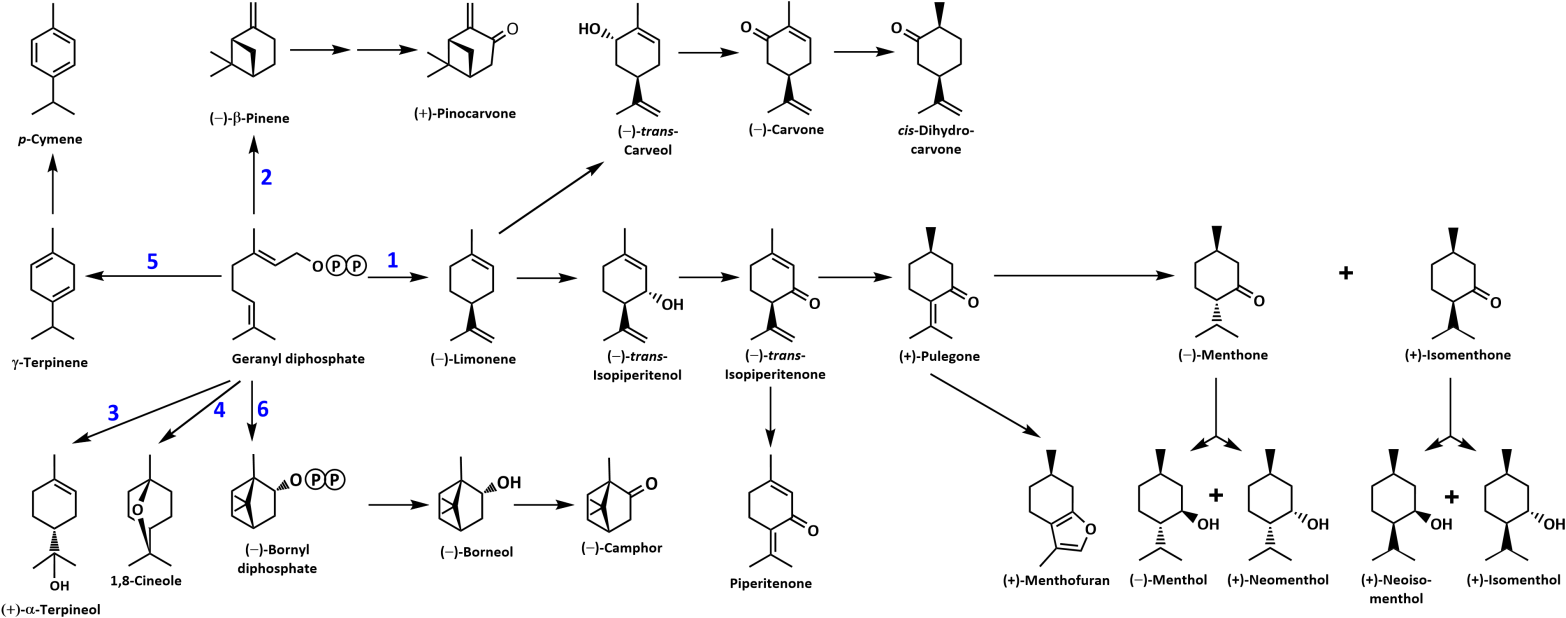
Overview of the mint monoterpene biosynthetic pathway. The circled letter P denotes a phosphate moiety. Reactions catalyzed by MTSs are numbered: 1, (–)-limonene synthase; 2, (–)-β-pinene synthase; 3, (–)-α-terpineol synthase; 4, 1,8-cineole synthase; 5, γ-terpinene synthase; and 6, (–)-bornyl diphosphate synthase.

Members of the mint family are the source of valuable consumer products, including essential oils. Commonly grown cultivars in temperate climates are peppermint (*Mentha* ˣ *piperita* L.) and spearmint (*Mentha spicata* L.). The dominant monoterpenoids in these oils are derived from a common biosynthetic intermediate, (–)-limonene, but the pathway is branched, thereby leading to the accumulation of (–)-carvone in spearmint, while (–)-menthone and (–)-menthol are major constituents of peppermint (Lange, 2015a) (**Figure 1**). The Black Mitcham peppermint and Native spearmint cultivars, grown widely across the United States, are polyploid, sterile hybrids, and diploid ancestral species, such as horsemint (*Mentha longifolia* (L.) Huds.), have thus been advanced as model species for genetic analyses (Vining et al., 2005). Substantial chemical diversity of distilled oils has been reported for members of this genus but there is also considerable variation in the resistance to Verticillium wilt (Vining et al., 2005; Vining et al., 2022), caused by the soil-borne fungus *Verticillium dahliae* Kleb., the most destructive disease of mint in the United States (Dung, 2020). The fungus initially infects roots, subsequently invades the vascular system, and ultimately causes occlusion and, thus, restricted water movement (Daayf, 2015).

The commercial process for obtaining mint oils starts with cutting above-ground material and leaving it in the field to dry (Denny and Lawrence, 2007). The mint is then chopped and blown into a large container (mint tub) that sits on a trailer. The trailer is pulled to a still, where the tub is connected to a boiler *via* a hose at the bottom and to a condenser line at the top. Steam from the boiler is introduced into the tub and distributed through pipes on the tub floor. Volatilized mint constituents rise with the introduced steam and enter the condenser line, which carries the water-oil mixture through a series of water-cooled smaller lines, thus forming a condensate that is collected in a separator tank. The lower density oil forms a separate phase on top of the water and is drained into galvanized drums (Denny and Lawrence, 2007). Oil distillations can be performed on a laboratory scale in purpose-built glassware, such as a Likens-Nickerson apparatus, which was originally developed for capturing hop oil and uses two boiling flasks connected to a mixer/condenser (Likens and Nickerson, 1964). One flask is loaded with mint leaves in water, while the other contains an immiscible organic solvent (usually *n*-hexane) (Vining et al., 2019). Each flask is heated to the boiling point of the contained liquid, thus sending vapors along the arms of the apparatus to the central cold finger condenser where they are mixed and condensed. The lighter organic phase is then collected in a separatory trap with a stopcock (Likens and Nickerson, 1964). This approach collects volatiles in an organic solvent volume of typically 8 to 10 ml, of which only a small fraction (usually 1 µl) is injected onto a Gas Chromatograph (GC) for subsequent analysis. Mint leaves have anatomical structures called glandular trichomes that store copious amounts of volatiles and other constituents, and the substantial dilution of analytes by laboratory-scale distillation is thus acceptable for volatile analyses (Amelunxen, 1965; Amelunxen et al., 1969; Lange and Turner, 2013; Lange, 2015a). One main goal of the current study was to assess if organs other than leaves may produce volatiles that have not been reported to occur in mint before, thus enhancing our understanding of the chemical diversity. Roots, rhizomes, and stems have very small glandular trichomes or none (Kowalski et al., 2019) and volatile concentrations would thus be expected to be comparatively low. In such cases, trapping of volatilized metabolites from the headspace above samples, enabled by techniques such as Solid Phase Microextraction (SPME), is generally more suitable (Tholl et al., 2006). Several studies have demonstrated that SPME is a reliable method for mint volatile analysis (Rohloff, 1999; Cordero et al., 2012; Silva and Câmara, 2013; Taherpour et al., 2017), and we thus developed a SPME-based approach for assessing volatiles in selected mint species.

To further explore chemical diversity, we assessed if the inoculation of roots with *V. dahliae* has effects on the volatile repertoire of mint organs relevant for the early stages of infection (roots, rhizomes, and stems). It is well known that mint-fungal interactions, both beneficial and detrimental, can have dramatic effects on the production of terpenoid volatiles (Karagiannidis et al., 2011; Arango et al., 2012; Bharti et al., 2013; Johnson et al., 2013; del Rosario Cappellari et al., 2015; Urcoviche et al., 2015; Khalvandi et al., 2019; Merlin et al., 2020; Khalvandi et al., 2021). The interactions between mint and fungi can be mutualistic (e.g., the mycorrhizal fungus *Glomus fasciculatum*) (Gupta et al., 2002), endosymbiotic (Mucciarelli et al., 2007) or pathogenic (Dung, 2020). Following the analysis of volatiles released by different organs of mint plants grown under various conditions, we took advantage of the recently completed chromosome-level assembly of the *M. longifolia* CMEN 585 genome and published transcriptome data sets for several mint species (Ahkami et al., 2015; Vining et al., 2017; Chen et al., 2021; Vining et al., 2022) to identify candidate genes coding for monoterpene synthases (MTSs), the enzymes that catalyze the first committed step of monoterpenoid volatile biosynthesis. The products generated by these as yet uncharacterized MTSs, in conjunction with those characterized previously, can explain the volatile profiles observed in our experiments.

## Materials and methods

### Plant materials and greenhouse growth conditions

Peppermint (Black Mitcham cultivar) and spearmint (Native cultivar) were supplied by the Mint Industry Research Council (Salem, OR, USA). *M. longifolia* accessions CMEN 585 (PI 557767) and CMEN 584 (PI 557769) were obtained from the U.S. Department of Agriculture National Clonal Germplasm Repository (Corvallis, OR, USA). Greenhouse-grown plants were maintained under the following conditions: temperature at 23–27°C; humidity at 60–75%; natural lighting supplemented with sodium vapor lights to generate a consistent 16 h day/8 h night cycle; average light intensity during the day was 250 μE m^−2^ s^−1^; daily watering and fertilizer treatment once a week with Peters 20/20/20 (R.J. Peters Inc., Chesterfield, MI, USA).

### Growth chamber growing conditions for *V. dahliae* inoculations and mock-inoculated controls

Plant material for cultivation in growth chambers was established from 6-10 cm cuttings taken off apical and auxiliary stems of pre-flowering greenhouse-grown plants. Cuttings were placed in plastic trays filled with autoclaved Sunshine Mix #1-Fafard-1P (Sun Gro Horticulture; Agawam, MA, USA), moistened with autoclaved double-distilled water, and the trays placed in plastic flats with an 8 cm humidity dome. Trays were moved to a E-75L1 growth chamber (Percival Scientific, Perry, IA, USA) fitted with a programmable light timer (Woods Model # 59745, supplied by Coleman Cable, Waukegan, IL, USA). The growth chamber was set at 22°C, 10 h light/14 h dark for peppermint and spearmint; 22°C, 8 h light/16 h dark for *M. longifolia* accessions CMEN 584 and CMEN 585 (average light intensity during illumination was 150 μE m^−2^ s^−1^). Flats were rotated daily and moistened with autoclaved, double-distilled water. The 8 cm humidity dome was replaced with a 20 cm humidity dome after one week. Humidity domes were removed completely after two weeks, and plants were not watered the day before performing root dips to aid in soil removal from roots. The *V. dahliae* strain used in our experiments had been collected from infected mint fields in Oregon (Vining and Pandelova, 2022). Fungal suspensions were initiated by placing a 1 cm^2^ agar plug into 125 ml of liquid Czapek-Dox broth (PhytoTechnology Laboratories, Lenexa, KS, USA). The liquid cultures were placed in a designated shaker at 180 rpm and 23°C for 7 – 9 days. Fungal suspensions for inoculation were prepared by filtering the culture through autoclaved Miracloth (Calbiochem, La Jolla, CA, USA) using an autoclaved Büchner funnel and flask setup with a UN810 FTP Laboport vacuum pump (KNF Neuberger, Trenton, NJ, USA). The filtered conidia suspension was evenly distributed across centrifuge tubes and spun down at 10,000 x *g* for 5 min. Autoclaved, double-distilled water was added to the pellets, mixed, and diluted to a final concentration of 10^7^ conidia per ml solution (determined by use of a Bright-Line hemacytometer (Hausser Scientific, Horsham, PA, USA) under a light microscope (DM LM, Leica Microsystems, Wetzlar, Germany). Root dips were performed by shaking off soil from plant roots and dipping roots into glass jars of either 10 ml autoclaved, double-distilled water (controls) or 10 ml *V. dahliae* suspension (experimental treatment). Roots were held in the jars for 5 min, before being planted in new plastic trays prefilled with soil as described above. Control and experimental treatment flats were placed on the same growth chamber platform and rotated daily for 6 d before harvesting (growing conditions as outlined above). Plants were watered sparingly to facilitate inoculation.

### Harvest of plant material and capturing volatiles by hydrodistillation

Leaves were picked from stems, avoiding the top and bottom nodes (approximately 3 g fresh weight). Hydrodistillations were performed in a modified Likens-Nickerson apparatus for 60 min using *n*-hexane as carrier solvent based on a previously published method (Ringer et al., 2003). An aliquot of the *n*-hexane fraction, which contained the volatile oil constituents, was transferred to a 2-mL glass vial. A 1 ml aliquot of this extract was analyzed by Gas Chromatography – Mass Spectrometry (GC–MS) as described under “Separation, Detection and Analysis of Volatiles”.

### Harvest of plant material, trapping of volatiles by static headspace SPME, and analysis of volatiles by GC–MS

Plants were removed from soil and briefly shaken. Rhizomes and roots were rinsed with autoclaved double-distilled water before separating them with scissors. Additional portions of soil were removed from roots with tweezers, then rinsed again, and gently dabbed dry between two sheets of clean paper towels to remove excess water. Rhizome segments of 0.5 cm were cut away from the lowest portion of stem and dabbed on a separate paper towel to remove excess water. Stem segments of 0.5 cm were cut with scissors at 2.5 cm above the soil line, avoiding nodes. Scissor blades and tweezer tips were wiped down using Kimwipes (Kimberly-Clark-Kimtech, Irving, TX, USA) first wetted with 70% EtOH, then wiped down again using Kimwipes saturated with autoclaved double-distilled water. Oil samples from leaves were taken by gently rubbing the surfaces of three leaves with a single 2.5 cm x 1 cm piece of clean paper towel. This was necessary because the quantity of volatiles released from leaves was very high and quickly saturated the SPME fiber. All plant tissue samples were weighed in pre-tared 20 ml screw-thread glass vials (Microliter Analytical Supplies, Suwanee, GA, USA), while the paper leaf rubbings were loosely folded and dropped into untarred vials. All vials were sealed within 60 seconds using 16 mm screw-top caps (butyl rubber septa with polytetrafluoroethylene coating (Wheaton MicroLiter; septa were prebaked overnight at 150°C). A Leap Technologies Combi PAL autosampler was outfitted with SPME syringe adapter, agitator module, and fiber conditioning station (CTC Analytics AG, Zwingen, Switzerland). SPME fiber assemblies were 23 gauge 65 µm PDMS/DVB StableFlex (Supelco; Bellefonte, PA, USA) used in conjunction with a Merlin Microseal High Pressure Nut Kit (Restek, Bellefonte, PA, USA) and 0.75 mm id Ultra Inert splitless liner with SPME taper (Agilent Technologies, Inc.; Santa Clara, CA, USA). Samples were preincubated at 60°C in the agitator (rotating at 250 rpm) for 10 min, before a SPME fiber was inserted (vial penetration of 22 mm) and exposed to headspace volatiles for 15 min. The SPME fiber was then inserted into the GC inlet at an injection depth of 54 mm and volatiles were desorbed for 10 s at 220°C in splitless injection mode, followed by a purge step (1 min at 20 ml/min) (6890N system; Agilent Technologies, Santa Clara, CA, USA). The fiber was reconditioned under a stream of N_2_ for 3 min at 250°C right after the injection.

### Separation, detection and analysis of volatiles

The separation of headspace volatiles was achieved over a DB-5MS column (30 m ˣ 0.25 mm, 0.25 µm film thickness; J&W Scientific, Folsom, CA, USA) at a flow rate of 1 ml/min He. The oven program started at 165°C, with a ramp of 25°C/min to 215°C, and a final hold at this temperature for 3 min. Through a transfer line, the column end reached into the ion source of a quadrupole MS detector (5973 *inert* system; Agilent Technologies, Santa Clara, CA, USA). The ion source was set to 230°C with an ionization voltage of 70 eV (electron ionization). The scan range was set to 40 – 350 *m/z*. The plant material after volatile capture was dried in an oven at 50°C overnight and weighed to determine the dry weight. Chiral analyses were performed on the same GC–MS system using a Cyclodex-B column (30 m ˣ 0.25 mm, 0.25 µm film thickness; J&W Scientific, Folsom, CA, USA). The following GC settings were used: injector temperature 250°C; injector split 1:20; sample volume 1 µl; flow rate 2 ml/min with He as carrier gas; oven heating program with an initial ramp from 40°C to 120°C at 2°C/min, followed by a second ramp to 200°C at 50°C/min, and a final hold at 200°C for 2 min. Identification of volatiles was achieved by matching retention times and target ions to those of authentic standards.

Data analysis was performed with the ChemStation E.02.00.493 software (Agilent Technologies, Santa Clara, CA, USA), with peak annotation and relative quantitation being achieved based on a combination of i) ion extraction *via* ChemStation’s QEdit function, ii) a dedicated spectral library for volatiles (Adams, 2007), iii) the NIST 20 library (National Institute of Standards and Technology, Gaithersburg, MD, USA), and iv) an extensive in-house library of authentic standards for volatiles (purchased from commercial sources, isolated from plant oils or obtained by chemical synthesis; the identity of all compounds in this library has been confirmed using GC–MS and nuclear magnetic resonance spectroscopy). The QEdit function in MSD ChemStation was specifically used to identify peaks of closely eluting compounds using pre-defined retention times and m/z values. Obtaining absolute values (ng per g tissue or similar) when acquiring static headspace SPME data is extremely challenging and only possible for a small number of selected analytes. It would require optimization, for each individual analyte, of fiber affinity, sensitivity, reproducibility, and linear range; for complex matrices, one would also have to assess competition for all analytes; additional parameters for optimization would be the extraction time and temperature for each analyte; finally, data for an external standard curve would have to be acquired for each analyte present in a matrix that mimics the samples but does not contain the analyte (Roberts et al., 2000). Since the goal of the current study was to assess chemical diversity (which includes a larger number of analytes), we chose to employ a simplified quantitation approach. Tissue used for volatile analyses was subsequently dried (48 h at 60°C) and the weight determined, which allowed the quantitation of analytes as peak abundance per unit weight, which is comparable across samples and the closest we can get to absolute quantitation within the scope of the study. Statistical analyses were performed using a two-tailed Mann-Whitney U test in Excel (Microsoft 365, Redmond, WA, USA).

### Phylogenetic analysis of MTSs

The sequences of functionally characterized MTSs from Lamiaceae were obtained from the National Center for Biotechnology Information (NCBI) protein database. MTS candidates of *M. longifolia* CMEN 585 had been identified previously based on the recently completed chromosome-level genome assembly (Chen et al., 2021; Vining et al., 2022). One MTS candidate from *M.* ˣ *piperita* (Black Mitcham cultivar) was identified by searching the translated transcriptome consensus assembly for leaf glandular trichomes of this species (NCBI Short Read Archive BioProject Identifier PRJNA 245825) against functionally characterized 1,8-cineole synthases of the Lamiaceae with a 60% sequence identity cutoff (sequences provided in **Supplemental Table S1**). The evolutionary history of MTSs was inferred by using the Maximum Likelihood method and JTT matrix-based model (Jones et al., 1992). The bootstrap consensus tree inferred from 1,000 replicates (Felsenstein, 1985) was taken to represent the evolutionary history of the taxa analyzed. Branches corresponding to partitions reproduced in less than 50% bootstrap replicates were collapsed. Initial trees for the heuristic search were obtained by applying the Neighbor-Joining method to a matrix of pairwise distances estimated using the JTT model (Jones et al., 1992). This analysis involved 40 amino acid sequences (**Supplemental Table S1**). Evolutionary analyses were conducted in the MEGA software (version 11) (Tamura et al., 2021).

### Functional characterization of MTSs

Synthetic cDNAs corresponding to candidate transcripts Ml_15999 and Ml_14850 (excluding plastidial targeting sequences) were obtained from Twist Bioscience (San Francisco, CA, USA or Biomatik (Wilmington, DE, USA), while the cDNA corresponding to candidate Ml_38055 (also excluding plastidial targeting sequence) was amplified from first-strand cDNA representing transcripts present in isolated *M. longifolia* CMEN 585 glandular trichomes (forward primer: ATGCGACGATCCGGAAATTACAAACCTAC; reverse primer: CTAAACAATAGGTTCAAATATGAGACCTAAAATG). A synthetic gene corresponding to peppermint candidate Mp_12413 (excluding plastidial targeting sequence) was ordered from Twist Bioscience (San Francisco, CA, USA). The accession numbers of all cDNAs are provided at the end of this article. All genes were integrated into the multiple cloning site of the pET28b expression vector (Novagen, Madison, WI, USA). Plasmids were transformed into chemically competent cells of *E. coli* strain BL21, which were then grown in 5 ml of liquid LB medium and move to 150 ml culture at 37°C with shaking at 350 rpm (Series 25, New Brunswick, ThermoFisher Scientific, Waltham, MA, USA) to an OD_600_ of 0.8. Expression of MTS genes was induced with 0.5 mM isopropyl β-D-1-thiogalactopyranoside (GoldBio, St. Louis, MO, USA) and cells were grown for another 24 h at 16°C and 350 rpm (Series 25, New Brunswick, ThermoFisher Scientific, Waltham, MA, USA). Bacterial cells were harvested by centrifugation at 5,000 x *g* and resuspended in 300 ul MOPSO buffer, pH 7.0, supplemented with 1 mM dithiothreitol (DTT; GoldBio, St. Louis, MO, USA). Cells were lysed using a model 475 sonicator (VirTis, Gardiner, NY, USA), with three 15 s bursts and cooling on ice for 45 s between bursts. The resulting homogenate was centrifuged at 15,000 x *g* for 30 min at 4°C, and recombinant protein present in the supernatant purified over Ni^2+^ affinity columns according to the manufacturer’s instructions (Marvelgent Biosciences, Canton, MA, USA). *In vitro* assays were performed in 2-ml glass vials containing 200 µg purified enzyme in MOPSO buffer (50 mM) containing DTT (1 mM) and MgCl2 (50 mM) (total volume of reaction mixture 100 µl). A prenyl diphosphate substrate (geranyl diphosphate, neryl diphosphate, (*E,E*)-farnesyl diphosphate or (*Z,Z*)-farnesyl diphosphate) was added to a final concentration of 0.5 mM. The assay mixtures were overlaid with 100 µl *n*-hexane (containing 15 ng/μL camphor as internal standard for subsequent analyses) (Avantor, Center Valley, PA, USA) and incubated at 30°C for 16 h on a multi-tube rotator (Labquake, Barnstead Thermolyne, Ramsey, MN, USA). Following addition of 20 μL of a solution containing 3 units of potato apyrase and 0.5 units of wheat germ acid phosphatase (SigmaAldrich, St. Louis, MO, USA) in 1 M sodium acetate (pH 5.0), the mixture was rotated for another 24 h at 31°C. To assess if bornyl diphosphate was generated as a product (which was then hydrolyzed to borneol by the addition of apyrase and acid phosphatase) or borneol was produced directly, we also performed assays without the addition of apyrase and acid phosphatase (no borneol was detected). Furthermore, negative control assays with boiled enzyme extracts were performed (no band visible on gels, no activity detected). Enzymatic reactions were stopped by vigorous mixing of the contents of the tubes, followed by 30 min at -20°C for phase separation (aqueous phase solidifies and organic phase remains liquid on top). The organic phase was removed and transferred to glass vial inserts and stored in GC vials at -20°C until further analysis. Assay products were analyzed by chiral GC analysis as described previously (Srividya et al., 2016). The composition of assay products was determined based on calibration curves obtained with authentic standards.

## Results

### Pilot experiment and experimental design considerations

To test the suitability of static headspace SPME for the analysis of mint volatile diversity, we performed a pilot experiment using greenhouse-grown plants. Leaves were subjected to hydrodistillation (*n*-hexane as carrier solvent) with subsequent analysis by GC–MS. The extract obtained after hydrodistillation was processed in one of two ways: i) direct injection onto the GC or ii) placing an aliquot of the extract in a glass vial to allow volatiles released into the headspace to be absorbed by a SPME fiber, which was then inserted into the GC inlet for volatile desorption. Representatives of four accessions were analyzed in this pilot experiment: peppermint (Black Mitcham cultivar), spearmint (Native cultivar), and *M. longifolia* accessions CMEN 585 and CMEN 584. The profile of the main terpenoid volatiles was generally very similar across these samples (**Table 1** and **Supplemental Table S2**). (–)- Menthone and (+)-menthofuran were the most prominent constituents of oils extracted from peppermint (55 – 58% and 14 – 16%, respectively), (–)-carvone was the principal volatile of spearmint and *M. longifolia* CMEN 584 oils (79 – 84% and 70 – 81%, respectively), and (+)-pulegone was the most abundant terpenoid of *M. longifolia* CMEN 585 oils (69 – 74%). However, a substantially lower amount of germacrene D (< 1%) was detected by headspace SPME when compared to the directly injected liquid extract (5%) in *M. longifolia* CMEN 585 (**Table 1** and **Supplemental Table S2**), indicating that SPME introduces a bias against higher-boiling sesquiterpenes, which are only minor constituents of the mint species of interest in the present study. A third sample type was generated by gently rubbing leaves against a rectangular piece of paper towel, which was then placed in a glass vial, and volatiles released into the headspace were trapped by SPME. This adjustment was necessary because even the smallest quantity of leaf material we could weigh consistently (0.5 mg) emitted quantities of volatiles that exceeded the capacity of the SPME fiber. Distilled peppermint extracts measured *via* SPME had a lower quantity of (–)-menthone (58% versus 64%) and higher amount of (+)-menthofuran (16% versus 0.3%), and a lower amount of (–)-menthol (5% versus 13%) compared to those processed by static headspace SPME of leaf rubs (**Table 1**). (–)-Limonene was generally higher in distilled samples (all accessions). (+)-Pulegone levels were lower in distilled samples of *M. longifolia* CMEN 585 (74% versus 90%) (**Table 1**). While the relative percentages of volatiles differed between extracts and headspace samples, each tested method was highly reproducible (**Supplemental Table S2**); because our main goal was to investigate the chemical diversity of volatile blends (which was captured by all methods), we thus proceeded with headspace SPME to allow direct comparisons across organs.

**Table 1:** Terpenoid profiles of mint leaves observed when employing different volatile capturing methods (expressed as percent of total volatiles with standard error; n = 3-5).

Species	Terpenoid	Hexane Extract	Hexane Extract	Leaf Rub
(Cultivar/Accession)		(Direct Injection)	(SPME)	(SPME)
*Mentha* ˣ *piperita* L.	(-)-Menthone	54.7 ± 0.5	57.7 ± 1.6	63.5 ± 2.4
(Black Mitcham)	(+)-Menthofuran	14.4 ± 0.3	16.4 ± 0.5	0.3 ± 0.1
	(-)-Menthol	6.6 ± 0.1	4.7 ± 1.5	13.0 ± 1.3
*Mentha spicata* L.	(-)-Carvone	79.3 ± 0.9	83.6 ± 2.4	83.1 ± 8.5
(Native)	(-)-Limonene	7.7 ± 0.5	5.3 ± 1.3	2.5 ± 1.4
*Mentha longifolia* (L.) Huds.	(+)-Pulegone	68.9 ± 1.5	74.3 ± 5.4	90.1 ± 0.9
(CMEN 585)	Piperitenone	9.8 ± 0.5	3.4 ± 2.5	0.6 ± 0.1
	Germacrene D	5.2 ± 0.1	0.9 ± 0.6	0.2 ± 0.1
*Mentha longifolia* (L.) Huds.	(-)-Carvone	70.0 ± 1.0	80.5 ± 0.8	85.7 ± 1.7
(CMEN 584)	(-)-Limonene	14.1 ± 1.3	7.6 ± 1.2	2.8 ± 0.9
	(-)-Borneol	5.7 ± 0.1	4.6 ± 1.2	4.9 ± 1.3

For details see text. SPME, head space solid phase microextraction.

### Mint plants emit species- and organ-specific blends of terpenoid volatiles

**Figure 2:**
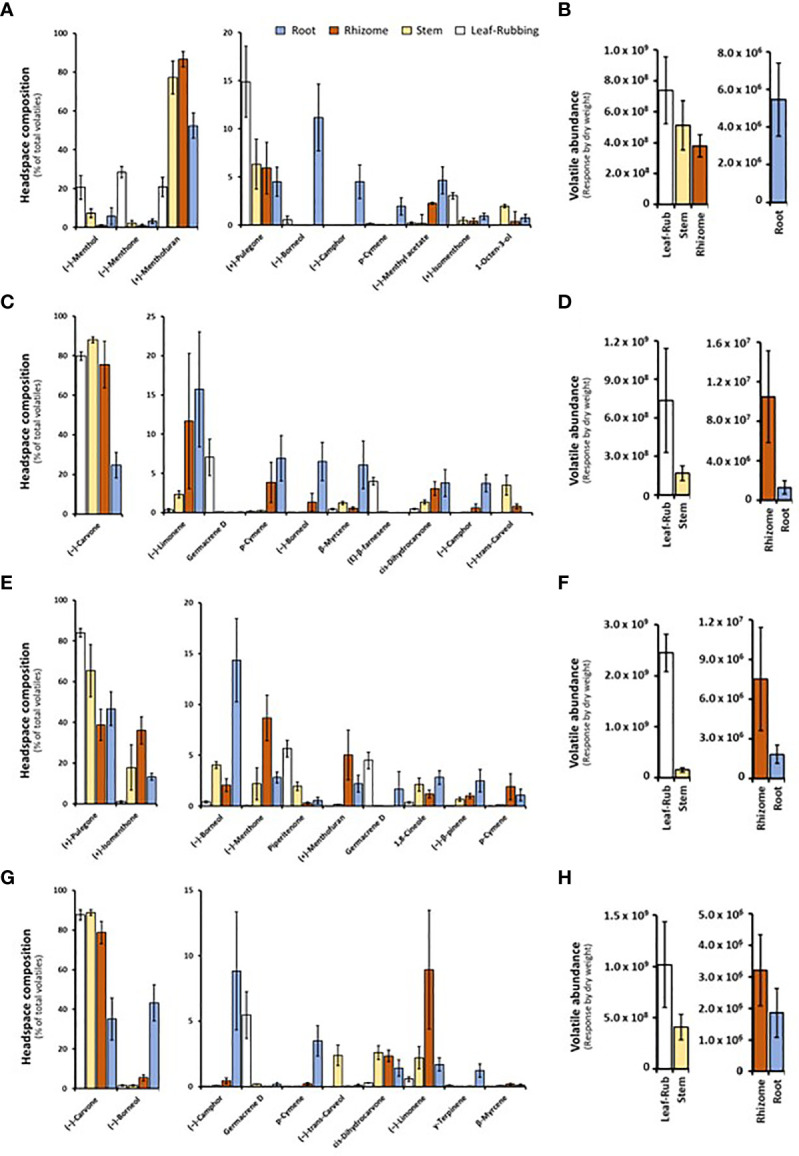
Monoterpenoid volatiles released by several organs of greenhouse-grown mint species as determined by SPME–GC–MS analysis (columns represent means of 6 biological replicates, with bars showing the standard error of the mean). **(A)** peppermint; **(C)** spearmint; **(E)** *M. longifolia* CMEN 585; and **(G)** *M. longifolia* CMEN 584. The volatile abundance is shown in **(B)** (peppermint), **(D)** (spearmint), **(F)** (*M. longifolia* CMEN 585), and **(H)** (*M. longifolia* CMEN 584). The color code of columns is as follows: white, leaves; yellow, stems; orange, rhizomes; and blue, roots.

Volatile bouquets emitted by stems and rhizomes from greenhouse-grown peppermint plants were characterized by high amounts of (+)-menthofuran (> 75% of detected volatiles), when compared to leaves, which released (–)-menthone (> 28%) and (–)-menthol as the most abundant monoterpenoids (> 20%) (**Figure 2A** and **Supplemental Table S3**). Root samples of peppermint had a unique volatile profile that, in addition to the presence of (+)-menthofuran (53%), was characterized by high amounts of (–)-borneol (11%), (–)-camphor (5%), (–)-menthyl acetate (5%), and (+)-pulegone (5%). Volatile emission from roots was particularly low compared to all other sample types (**Figure 2B**).

(–)-Carvone was the most abundant monoterpenoid in the volatile blends emitted by spearmint plants across organs (≥ 80% in leaf rubs, stems, 76% in rhizomes; 25% in roots) (**Figure 2C** and **Supplemental Table S3**). Leaf samples also released sesquiterpenes (most prominently germacrene D (7%) and (*E*)-β-farnesene (4%)) as volatiles of note. Stem samples had (–)-*trans*-carveol and *cis*-dihydrocarvone as additional noteworthy, volatilized metabolites. Rhizomes formed (–)-limonene (12%) and *p*-cymene (4%) as further prominent volatiles. The root volatile profile was more complex, with substantial contributions, in addition to (–)-carvone, coming from (–)-limonene (16%), *p*-cymene (7%), (–)-borneol (7%), and (+)-pinocarvone (6%) (**Figure 2C** and **Supplemental Table S3**). Volatile emission from leaf and stem samples was substantially higher than that of rhizomes and roots (**Figure 2D**).

The CMEN 585 accession of *M. longifolia* emitted (+)-pulegone as signature volatile from all samples (84% in leaves, 66% in stems, 39% in rhizomes, and 47% in roots) (**Figure 2E** and **Supplemental Table S3**). Other notable constituents of leaf volatile blends were piperitenone (6%) and germacrene D (5%). Stems released (+)-isomenthone (18%) and (–)-borneol (4%) as additional volatiles of note. The headspace above rhizome samples contained, in addition to (+)-pulegone, (+)-isomenthone (36%), (–)-menthone (9%), and (+)-menthofuran (5%) as abundant volatiles. Roots released (–)-borneol (14%) and (+)-isomenthone (13%) as noteworthy metabolites (in addition to (+)-pulegone) (**Figure 2E** and **Supplemental Table S3**). Volatile emission from leaves was substantially higher than that of all other sample types (**Figure 2F**).

The leaf profile of the *M. longifolia* accession CMEN 584 resembled that of spearmint, with (–)-carvone being dominant in the blends released by leaf rubbings (88%), stems (89%) and rhizomes (79%), with a slightly higher amount in roots (35%) (**Figure 2G** and **Supplemental Table S3**). The only other notable constituent in leaf surface oils was germacrene D (6%). Notable volatile constituents in rhizomes, aside from (–)-carvone, were (–)-limonene (9%) and (–)-borneol (5%). Stems emitted, in addition to (–)-carvone, smaller quantities of (–)-*trans*-carveol, *cis*-dihydrocarvone, and (–)-limonene (slightly above 2% each). The most abundant volatiles of roots were (–)-borneol (43%), (–)-carvone (35%), and (–)-camphor (9%) (**Figure 2G** and **Supplemental Table S3**). Volatile emission from leaf and stem samples was substantially higher than that of rhizomes and roots (**Figure 2H**).

In summary, our analyses by SPME–GC–MS showed substantial differences regarding volatiles emitted by different mint species, consistent with prior analyses using hydrodistillation (Ahkami et al., 2015; Vining et al., 2022). In addition, even within a species, each organ produced a unique blend of volatiles, which is novel information adding to our knowledge of the repertoire for volatile formation.

### Experimental conditions to assess the effects of *V. dahliae* inoculation increase volatile chemical diversity

Our previous analysis of the *M. longifolia* genome (accession CMEN 585) indicated the presence of genes coding for more than fifty terpene synthases (Chen et al., 2021). It is well known that the expression of terpene synthase genes is not only organ-, tissue, and cell type-specific, but they are also inducible by different environmental and experimental conditions (Degenhardt et al., 2009). To evaluate the inducibility of terpene emission, we designed a study to assess volatile blends released after inoculation of roots with the wilt pathogen *V. dahliae*. Two accessions with significant wilt resistance (spearmint and *M. longifolia* CMEN 585) and two susceptible accessions (peppermint and *M. longifolia* CMEN 584) were included in this work. The experiment was performed in climate-controlled growth chambers to ensure pathogen containment, necessitating growing conditions that were substantially different from those for the data sets acquired with greenhouse-grown plants as discussed above (see Materials and Methods for details). The experimental design also had to be modified substantially: i) plants were carefully taken out of their flats and soil shaken off; ii) roots were dipped in either sterilized water (controls) or a solution containing a known concentration of *V. dahliae* inoculum (experimental plants); and iii) plants were re-planted separately in pots containing sterilized soil.

**Figure 3:**
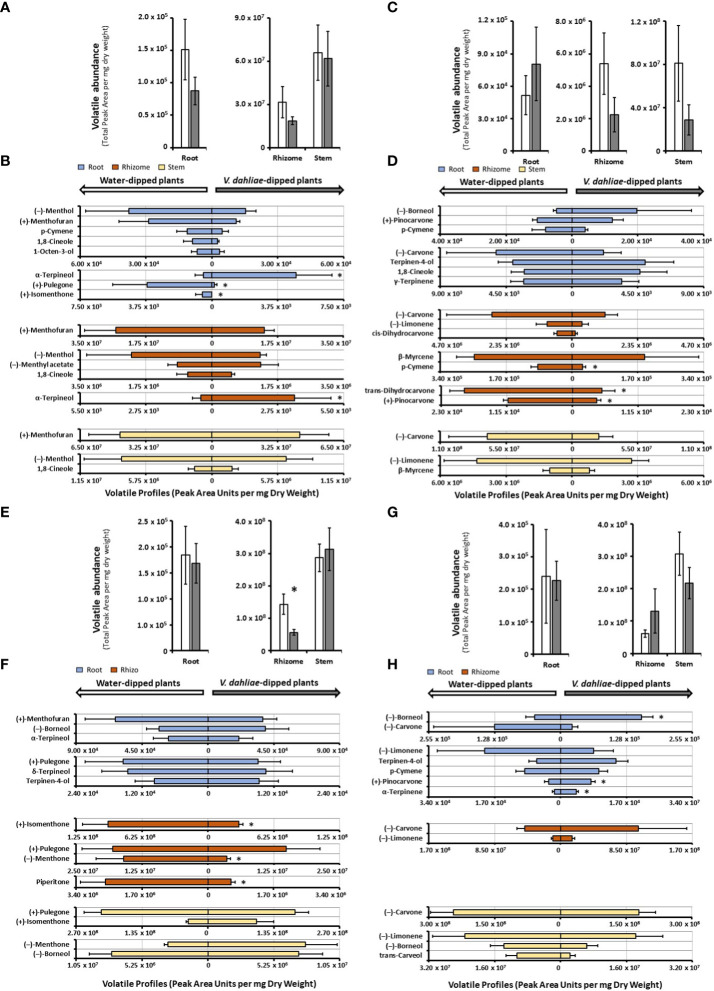
Monoterpenoid volatile profiles, as determined by SPME–GC–MS analysis, released by several organs of mint species maintained in growth chambers and prepared for *V. dahliae* inoculation (columns represent means of 4 – 6 biological replicates, with bars showing the standard error of the mean). The total volatile abundance of volatiles emitted by each organ is shown in panels **(A)** (peppermint), **(C)** (spearmint), **(E)** (M. longifolia CMEN 585), and **(G)** (M. longifolia CMEN 584, with white columns for controls and gray columns for experimental samples. The left panel of each butterfly diagram shows data for mock-inoculated controls, while the right panel has data for plants whose roots were inoculated with *V. dahliae*, with **(B)**, peppermint; **(D)**, spearmint; **(F)**, *M. longifolia* CMEN 585; and **(H)**, *M. longifolia* CMEN 584. The color code of columns is as follows: yellow, stems; orange, rhizomes; and blue, roots. An asterisk represent *P*-value < 0.5.

In mock-inoculated control samples, the release of volatiles from peppermint roots was markedly lower than that from rhizomes (roughly 200-fold less in both control and experimental samples) and volatile emission from rhizomes was roughly half of that of stems (**Figure 3A** and **Supplemental Table S4**). The emission from peppermint roots, rhizomes and stems was slightly reduced (not statistically significant) in *V. dahliae-*dipped experimental root samples when compared to the corresponding water-dipped controls (**Figure 3A**). The main constituents of volatile blends emitted by peppermint root controls were (–)-menthol (25% of total volatiles; 37,700 peak area units (PAU) per mg dry weight (DW)), (+)-menthofuran (18%), *p*-cymene (8%), 1,8-cineole (6%), and 1-octen-3-ol (4%) (**Figure 3B** and **Supplemental Table S4**). *V. dahliae* inoculation resulted in a slight reduction (not statistically significant) in the quantities of major emitted volatiles [(–)-menthol, (+)-menthofuran, *p*-cymene, and 1,8-cineole], dramatic reductions in the release of (+)-pulegone (25-fold from 3,698 to 139 PAU per mg DW; *P*-Value of 0.02) and (+)-isomenthone (from 549 PAU per mg DW to undetectable levels; *P*-Value of 0.02), and a striking 10-fold increase in the volatilization of (+)-α-terpineol (from 480 to 4,794 PAU per mg DW; *P*-value of 0.03) (**Figure 3B** and **Supplemental Table S4**). Peppermint rhizome controls emitted primarily (+)-menthofuran (83%; 2.5 **·** 10^7^ PAU per mg DW), with (–)-menthol (5%), (–)-menthyl acetate (3%), and 1,8-cineole (2%) as other noteworthy, volatilized constituents (**Figure 3B** and **Supplemental Table S4**). As a response to *V. dahliae* treatment, the release of the major constituents (+)-menthofuran and (–)-menthol decreased slightly (not statistically significant), while the emission of the minor component (+)-α-terpineol increased 7-fold (from 461 to 3,453 PAU per mg DW; *P*-value of 0.03). The mixture of volatiles released by peppermint stem controls was dominated by (+)-menthofuran (67%; 4.5 **·** 10^7^ PAU per mg DW), with (–)-menthol (9%), 1-octen-3-ol (9%), and 1,8-cineole (3%) being other noteworthy constituents (**Figure 3B** and **Supplemental Table S4**). No significant changes in volatile bouquets were detected when comparing water-dipped stem controls to the corresponding *V. dahliae*-inoculated samples.

The abundance of volatiles released by mock-inoculated spearmint root controls (5.2 **·** 10^4^ PAU per mg DW) was roughly 100-fold lower than that from rhizomes (5.4 **·** 10^6^ PAU per mg DW), and the volatile emission from rhizomes was 15-fold lower than that from stems (8.1 **·** 10^7^ PAU per mg DW) (**Figure 3C** and **Supplemental Table S4**). The overall quantity of volatiles increased slightly in *V. dahliae*-inoculated spearmint roots, while decreasing slightly in rhizomes and stems of experimental plants when compared to controls (not statistically significant) (**Figure 3C**). Roots of spearmint controls emitted (+)-pinocarvone as the most prominent volatile (26% of total volatiles; 10,519 PAU per mg DW), with *p*-cymene (13%), (–)-borneol (11%), terpinen-4-ol (10%), 1,8-cineole (7%), and γ-terpinene (7%) as other notable monoterpenes (**Figure 3D** and **Supplemental Table S4**). Slight increases (not statistically significant) in the emission of several volatiles [(-)-borneol,(+)-pinovarvone, terpinen-4-ol, and 1,8-cineole] were observed as a response to *V. dahliae* inoculation; a non-significant decrease of (-)-carvone was also detected. (–)-Carvone (35% of total volatiles; 2.9 **·** 10^6^ PAU per mg DW) was the most abundant volatile emitted by spearmint rhizome controls, with (–)-limonene (19%), *cis*-dihydrocarvone (15%), and β-myrcene (7%) being other notable constituents (**Figure 3D** and **Supplemental Table S4**). A *V. dahliae* treatment resulted in decreases in the release of *p*-cymene (3.3-fold from 88,424 to 26,991 PAU per mg DW *P*-Value 0.03), *trans*-dihydrocarvone (3.7-fold from 18,981 to 5,077 PAU per mg DW; *P*-Value 0.02), and (+)-pinovarvone (2.7-fold from 11,270 to 4,194 PAU per mg DW *P*-Value 0.02) compared to control samples. In headspace samples of spearmint stems (controls), (–)-carvone was dominant (84% of total volatiles; 7.1 **·** 10^7^ PAU per mg DW), with (–)-limonene (9%), *trans*-carveol and β-myrcene (both at < 2%) as other noteworthy contributors to volatiles blends(**Figure 3D** and **Supplemental Table S4**). A slight reduction (not statistically significant) in the concentrations of these volatiles were detected in stems of *V. dahliae*-inoculated samples (when compared to mock-inoculated controls).

The overall release of volatiles from mock-inoculated roots (1.8 **·** 10^5^ PAU per mg DW) of the *M. longifolia* CMEN 585 accession was 780-fold lower than from rhizomes (1.4 **·** 10^8^ PAU per mg DW), and volatile emission from rhizomes were 2-fold lower than those of stems (2.9 **·** 10^8^ PAU per mg DW) (**Figure 3E**). A *V. dahliae* treatment did not affect total root and stem volatiles significantly, but there was a 2.8-fold decrease in total volatile emissions from rhizomes when compared to those of corresponding controls (*P*-Value 0.03) (**Figure 3E**). The volatile blends emitted by *M. longifolia* CMEN 585 roots consisted of primarily (+)-menthofuran (37% of total volatiles; 63,416 PAU per mg DW), (–)-borneol (22%), and α-terpineol (13%), with (+)-pulegone (7%), δ-terpineol (7%), terpinen-4-ol (5%) as additional notable constituents (**Figure 3F** and **Supplemental Table S4**). There were no statistically significant differences between *V. dahliae*-inoculated and control root volatiles. The headspace above rhizome samples of *M. longifolia* CMEN 585 control plants was rich in (+)-isomenthone (65%), (+)-pulegone (14%), and (–)-menthone (11%). The quantities of three rhizome volatiles decreased as a response to *V. dahliae* inoculation when compared to controls: (+)-isomenthone was reduced by 3.2-fold (from 9.5 **·** 10^7^ to 2.9 **·** 10^7^ PAU per mg DW; *P*-Value 0.01), (–)-menthone was 4.4-fold lower (from 1.6 **·** 10^7^ to 3.6 **·** 10^6^ PAU per mg D; *P*-Value 0.03), and the concentration of piperitone was decreased 4.4-fold (from 2.6 **·** 10^6^ to 5.9 **·** 10^5^ PAU per mg DW; *P*-Value 0.01). Stems of *M. longifolia* CMEN 585 control samples emitted primarily (+)-pulegone (75%) and (+)-isomenthone (15%), with (-)-borneol (3%) and (–)-menthone (1%) being the only other noteworthy constituents (**Figure 3F** and **Supplemental Table S4**). A *V. dahliae* treatment resulted in slight increases (not statistically significant) in the emission of (+)-isomenthone, (–)-menthone and (–)-borneol from stems.

The release of volatiles from roots of *M. longifolia* accession CMEN 584 (2.4 **·** 10^5^ PAU per mg DW) was 255-fold lower than that from rhizomes (6.1 **·** 10^7^ PAU per mg DW), and volatile emission from rhizomes was 5-fold less than from stems (3.1 **·** 10^8^ PAU per mg DW) (**Figure 3H**). The experimental *V. dahliae* treatment caused a slight increase of volatile emissions from rhizomes and a slight decrease of volatile release from stems (not statistically significant), and no changes in roots (**Figure 3G** and **Supplemental Table S4**). Roots emitted a volatile blend rich in (–)-borneol (33% of total volatiles; 1,208,008 PAU per mg DW) and (–)-carvone (24%), with (–)-limonene (9%), *p*-cymene (6%), terpinen-4-ol (4%), and *cis*-dihydrocarvone (4%), (+)-pinocarvone (2%), and α-terpinene (1%). A *V. dahlia*e treatment-induced increase in the emission of (-)-borneol (3.1-fold; *P*-Value 0.02), (+)-pinocarvone (2.6-fold; *P*-Value 0.03), and α-terpinene (2.5-fold; *P*-Value 0.02) was observed. The dominant monoterpenoid emitted from rhizomes of control plants was (–)-carvone (75%), with only (–)-limonene (18%) being another substantial contributor to the profile (all other constituents at < 1%). *V. dahliae* inoculation led to slight increases (not statistically significant) in (–)-carvone and (–)-limonene release. Stems of controls emitted high quantities of (–)-carvone (80%; 2.4 **·** 10^8^ PAU per mg DW) and lower amounts of (–)-limonene (8%), (-)-borneol (4%), and *trans*-carveol (3%) (**Figure 3G** and **Supplemental Table S4**). Slight decreases (not statistically significant) in the quantities of these metabolites were observed as a response to *V. dahliae* inoculation.

### Cloning and functional characterization of candidate MTSs involved in mint terpenoid volatile emissions

**Figure 4:**
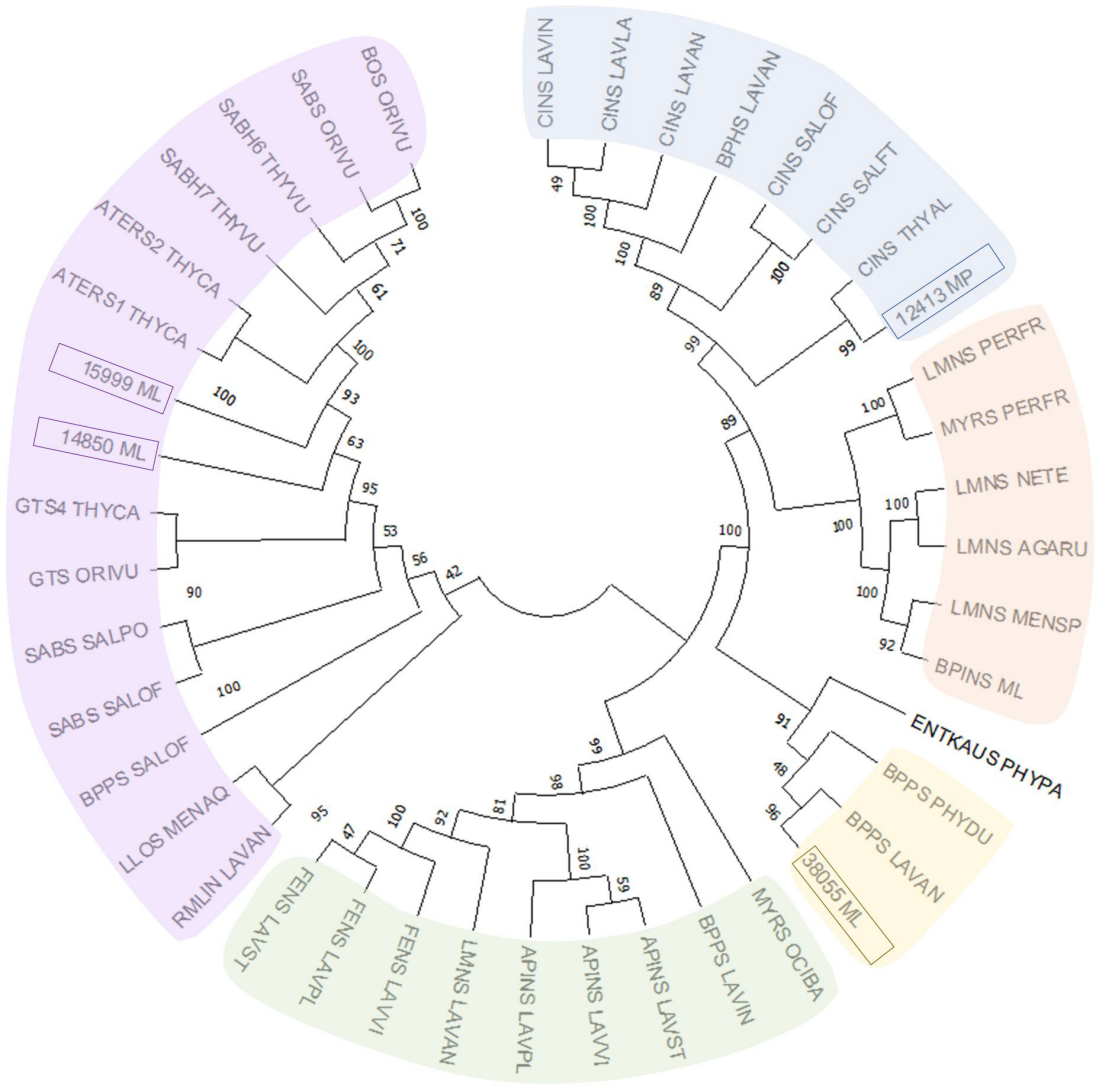
Evolutionary analysis of characterized MTSs of the Lamiaceae by the Maximum Likelihood method. Methodological details are provided in Materials and Methods, while sequences and abbreviations for MTSs are given in **Supplemental Table S1**.

Despite the large body of published work on the biosynthesis of functionalized monoterpenoids in mint (Lange and Srividya, 2019), surprisingly, only two MTSs have been functionally characterized: (–)-limonene synthase from spearmint (Colby et al., 1993) and (–)-β-pinene synthase from *M. longifolia* CMEN 585 (Kim et al., 2022). These MTSs are involved in the biosynthesis of *p*-menthane monoterpenoids, such as (–)-menthol of peppermint, (–)-carvone of spearmint, and (+)-pulegone of *M. longifolia* CMEN 585 (all derived from (–)-limonene) and (+)-pinocarvone (derived from (–)-β-pinene) (**Figure 1**). However, MTSs must also exist to produce other volatiles detected in our experiments, including α-terpineol, 1,8-cineole, *p*-cymene (through γ-terpinene as precursor), as well as (–)-borneol and (–)-camphor (both through (–)-bornyl diphosphate as precursor) (**Figure 1**). To identify candidate genes coding for these MTSs, we had taken advantage of the recently published chromosome-level assembly of *M. longifolia* accession CMEN 585 and performed subsequent bioinformatic analyses (Chen et al., 2021; Vining et al., 2022). Translated peptide sequences of candidate genes with high identity to previously characterized MTSs across the Lamiaceae (mint family) were then subjected to a phylogenetic analysis. *M. longifolia* CMEN 585 candidate Ml_15999 occupied a clade with previously characterized α-terpineol synthases of Cretan thyme (*Thymus caespititius* Brot.) (Lima et al., 2013) (referred to as ATERS1 and ATERS2 in **Figure 4**). Candidate Ml_14850 clustered with γ-terpinene synthases of the genera *Thymus* and *Origanum* (Mendes et al., 2014; Tohidi et al., 2020) (labeled as GTS4 and GTS, respectively, in **Figure 4**). Candidate Ml_38055 formed a clade with previously characterized bornyl diphosphate synthases of *Lavandula angustifolia* Mill. and *Phyla dulcis* (Trevir.) Moldenke (Despinasse et al., 2017; Hurd et al., 2017) (indicated as BPPS in **Figure 4**). Candidate Mp_12413 from peppermint clustered with 1,8-cineole synthases (labeled as CINS in **Figure 4**) of several species of the Lamiaceae (Wise et al., 1998; Kampranis et al., 2007; Demissie et al., 2012; Ali et al., 2022).

**Figure 5:**
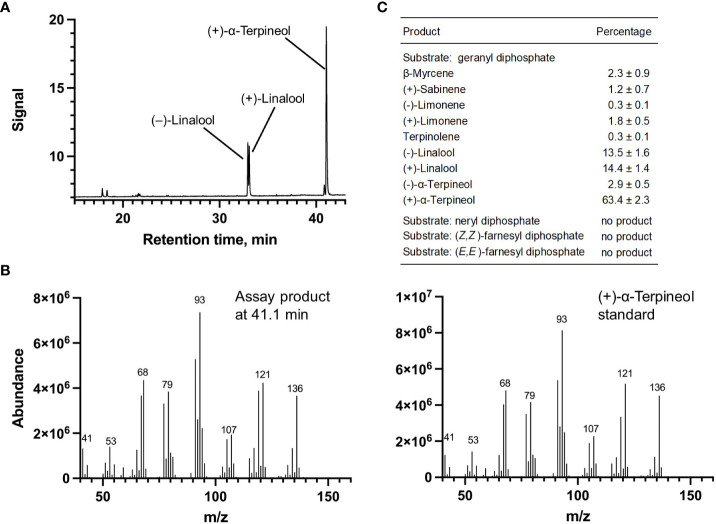
Characterization of *M. longifolia* CMEN 585 candidate Ml_15999. **(A)** GC chromatogram with annotation for major peaks; **(B)** mass spectrum of the main enzymatically generated product and the matching authentic standard [(+)-α-terpineol); and **(C)** tabulated product profile.

**Figure 6:**
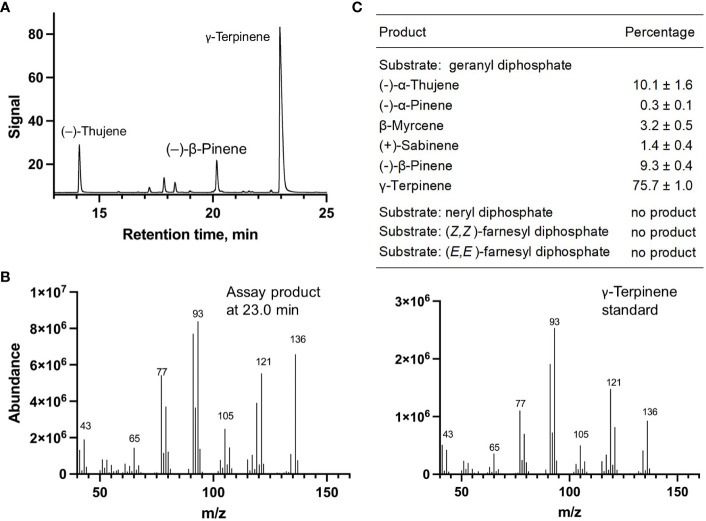
Characterization of *M. longifolia* CMEN 585 candidate Ml_14850. **(A)** GC chromatogram with annotation for major peaks; **(B)** mass spectrum of the main enzymatically generated product and the matching authentic standard (γ-terpinene); and **(C)** tabulated product profile.

**Figure 7:**
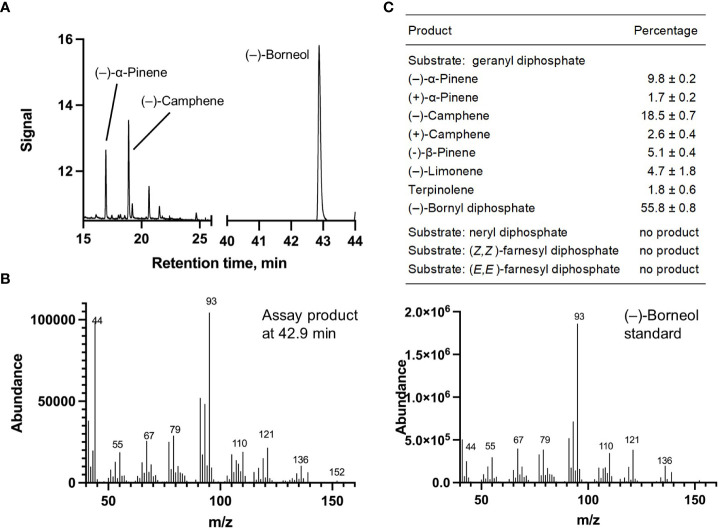
Characterization of *M. longifolia* CMEN 585 candidate Ml_38055. **(A)** GC chromatogram with annotation for major peaks; **(B)** mass spectrum of the main enzymatically generated product (following hydrolysis) and the matching authentic standard [(–)-borneol, which is hydrolytically released from bornyl diphosphate]; and **(C)** tabulated product profile.

**Figure 8:**
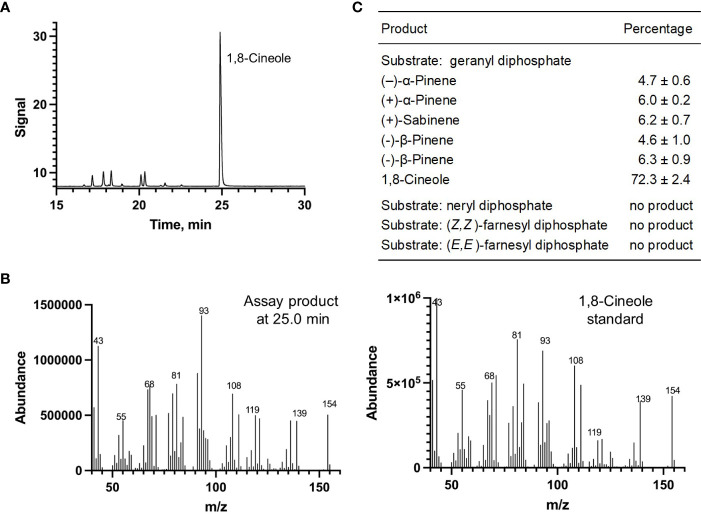
Characterization of *M. longifolia* CMEN 585 candidate Mp_12413. **(A)** GC chromatogram with annotation for major peaks; **(B)** mass spectrum of the main enzymatically generated product and the matching authentic standard (1,8-cineole); and **(C)** tabulated product profile.

Each of the four candidate cDNAs was cloned into an expression vector (pET 28b), the construct transformed into *Escherichia coli*, transgene expression induced, the recombinant protein purified, a functional assay performed, and products were analyzed using a GC equipped with a flame ionization detector (as described in Srividya et al., 2016). The dominant product (63%) of Ml_15999 from *M. longifolia* was (+)-α-terpineol (> 95 enantiomeric excess (ee)), with (–)-linalool and (+)-linalool as prominent side-products (each roughly 14%) (**Figures 5A–C**). Ml_14850 of *M. longifolia* generated γ-terpinene as primary product (76%), with (–)-β-pinene (9.3%) and (–)-thujene (10%) as notable byproducts (**Figures 6A–C**). The principal product of Ml_38055 was (–)-bornyl diphosphate (56%; detected as (-)-borneol after hydrolysis at >99% ee), with (–)-camphene (18.5%), (–)-α-pinene (10%), and (–)-β-pinene (5%) as significant side-products (**Figures 7A–C**). 1,8-Cineole was detected in the volatile blends emitted by several peppermint samples (**Figure 3**), and we thus searched previously published peppermint transcriptome data sets for additional MTS candidate genes. One of these candidates, Mp_12413, formed 1,8-cineole as main product (72%), with (+)/(–)-α-pinene (total of 11%), (+)/(–)-β -pinene (total of 11%) and (+)-sabinene (6%) as the most prominent side products (**Figures 8A–C**). All MTS candidates were assayed with neryl diphosphate, (*Z,Z*)-farnesyl diphosphate, and (*E,E*)-farnesyl diphosphate as alternative substrates, but none of these reactions were productive (**Figures 5C**, **6C**, **7C**, **8C**).

## Discussion

### Unique blends of volatiles are emitted by different mint organs

Most volatile analyses of mint species have been performed with oils obtained by steam distillation of above-ground material (Lawrence, 2006). Since a research-scale hydrodistillation that uses a carrier solvent is only suitable for analytes that are accumulated at fairly high levels in a tissue or organ of interest (Likens and Nickerson, 1964), we employed volatile capture by SPME as method to assess both above-ground and below-ground samples. These analyses indicated the presence of monoterpenoids that had previously been reported only as trace constituents with low annotation confidence. For example, *p*-cymene has occasionally (but not consistently) been reported as a 0.1% constituent of steam-distilled peppermint oil (Lawrence, 2008). We observed similar levels in both hydrodistilled and headspace SPME analyses of peppermint leaves, while the headspace above root samples of all accessions had a much higher proportion of this volatile (1.9% in peppermint, 6.9% in spearmint, 1.1% in *M. longifolia* CMEN 585, and 3.5% in *M. longifolia* CMEN 584). While trace quantities of (–)-borneol and (–)-camphor were observed in some leaf samples (but they were mostly undetectable), these volatiles were present at higher levels in volatile blends emitted from roots of peppermint (11 and 5%, respectively), spearmint (7 and 4%, respectively), and *M. longifolia* CMEN 584 (43 and 9%, respectively), while *M. longifolia* CMEN 585 roots emitted (–)-borneol (14%), but not (–)-camphor. To the best of our knowledge, neither borneol nor camphor had been described as mint constituents before. (+)-Pinocarvone, which has also not been reported as a mint oil constituent before, was detected in the headspace above spearmint root samples (6% of total volatiles).

The bulk of the mint leaf volatiles is synthesized and accumulated in specialized anatomical structures called peltate glandular trichomes, but such structures are not present on stems, rhizomes, or roots (Lange, 2015b). The formation of volatiles by all organs investigated as part of the current study may thus come as a surprise. However, it should be noted that the pathways for the formation of terpenoids and other volatiles are not restricted to glandular trichomes. The biosynthesis of volatiles in flowers often occurs in epidermal cells (Effmert et al., 2006). Volatiles can be produced throughout the leaf, as illustrated by the release of sesquiterpenes at the site of damage caused by herbivore feeding (Köllner et al., 2013). Terpenes can also be formed in many types of roots cells, as demonstrated for the formation of 1,8-cineole in *Arabidopsis thaliana* L. (Chen et al., 2004). We showed earlier that genes involved in monoterpene biosynthesis are responsive to treatment of *M. longifolia* with *V. dahliae* (Vining and Pandelova, 2022). Having demonstrated the formation of terpene volatiles by several mint organs in the present work, it will be interesting to investigate the localization of the relevant pathways outside glandular trichomes in future studies.

While the concentrations of volatiles emitted by mint roots were substantially lower than those released by stems and rhizomes, the discovery of unique blends formed in roots increases the known monoterpenoid repertoire of mint. This observation also begs the question if mint root volatiles might play roles in plant-plant and/or plant-microbe interactions, akin to evidence gathered as part of soil biome studies with other plants (Junker and Tholl, 2013; Peñuelas et al., 2014; Delory et al., 2016). The experiments discussed in the upcoming paragraph begin to address this question, but more work will be necessary in the future. The cloning of MTSs from mint that collectively generate the major root volatiles detected in our experiments (more details below) is an important first step to provide a better understanding of the molecular basis for volatile formation in mint organs other than leaves.

### Expansion of mint volatile portfolio through environmental and experimental conditions

A follow-up experiment was performed to assess if *V. dahliae* inoculation would induce the formation of volatiles not seen in control samples. Our design involved the removal of plants from sterile soil, inoculation of roots with a suspension containing fungal conidia (or water for control samples), and then reestablishing plants in new sterile soil. Plants were maintained in growth chambers to ensure fungal containment. Several differences were observed when comparing volatiles blends emitted from roots of mint plants maintained in greenhouses versus those that were kept in growth chambers and prepared for *V. dahliae* inoculation, in some cases even in control plants. For example, (–)-borneol and (–)-camphor were among the more abundant volatiles emitted by peppermint roots of greenhouse-grown plants, while *p*-cymene and 1,8-cineole were released at relatively high levels by roots of growth chamber-maintained plants. (–)-Carvone and (–)-limonene were the most prominent volatiles detected in the headspace above spearmint roots of greenhouse-grown plants, whereas (–)-borneol, (+)-pinocarvone, and *p*-cymene were the signature volatiles emitted by roots of growth-chamber-maintained plants. The most abundant volatiles released by roots of greenhouse-grown *M. longifolia* CMEN 585 were (+)-pulegone and (+)-isomenthone, while volatile blends emitted by roots of growth chamber-maintained plants were enriched in (+)-menthofuran, (–)-borneol, and α-terpineol. (–)-Camphor was among the more abundant volatiles in the headspace above roots from greenhouse-grown *M. longifolia* CMEN 584 plants, while (–)-limonene was released at much higher levels from roots of growth chamber-maintained plants. Interestingly, the overall volatile profiles emitted by rhizomes and stems were more comparable across all species and growth conditions investigated here, indicating that differences across root samples are more likely to result from the temporary removal of plants from the soil as an inevitable step for the experiment performed in growth chambers. Evaluating the differences in root volatile profiles in greenhouse-grown and growth chamber-maintained plants was a valuable exercise in the context of our project goal to evaluate terpenoid chemical diversity.

Statistically significant differences in volatile quantities released by plants whose roots were dipped in a *V. dahliae*-spore suspension (experimental) or dipped in water (controls) were evident in roots of mint species that are susceptible to *Verticillium* wilt disease (peppermint and *M. longifolia* CMEN 584), while rhizome volatiles were affected in species that show resistance to the disease (spearmint and *M. longifolia* CMEN 585). Our observations are certainly interesting, as volatiles could play roles in the communication between mint roots and the soil-borne *V. dahliae* pathogen. For example, soil treatments with terpene-containing formulations have been shown to reduce *V. dahliae* growth (Wei et al., 2016). Contrastingly, it has been shown that the overexpression of genes involved in monoterpene biosynthesis in transgenic *Arabidopsis thaliana* plants leads to an enhanced colonization by *V. longisporum*, a species closely related to *V. dahliae* (Roos et al., 2015). It is thus conceivable that specific volatile blends have functions in susceptibility or resistance to *Verticillium* wilt disease, which is an interesting hypothesis for further testing, but beyond the scope of the current study. In the present work, our emphasis was primarily to assess if experimental conditions could be used to increase the diversity of volatiles released by mint. While the quantities of volatile blends emitted by mint roots were low, the observed chemical diversity was surprising, and we thus embarked on efforts to characterize the genes underlying volatile production in mint, as discussed in the next paragraph.

### Mint MTSs are expressed in multiple organs, thus accounting for emitted volatiles

A (–)-limonene-forming MTS from spearmint (LMNS) had been cloned and characterized decades ago, with orthologs of peppermint and *M. longifolia* CMEN 585 having been characterized as well (Colby et al., 1993; Ahkami et al., 2015; Chen et al., 2021; Vining et al., 2022). LMNS generates the precursor for the main pathways active in peppermint, spearmint, *M. longifolia* CMEN 585, and *M. longifolia* CMEN 584, accounting for the formation of (–)-limonene, (–)-menthol, (–)-menthone, (–)-menthyl acetate, (+)-isomenthone, (+)-menthofuran, (+)-pulegone, piperitenone, (–)-*trans*-carveol, (–)-carvone, and *cis*-dihydrocarvone (**Figures 1**, **2**). LMNS is expressed at high levels in leaf glandular trichomes of all mint species investigated thus far (Lange et al., 2000; Ahkami et al., 2015; Vining et al., 2022). In *M. longifolia* CMEN 585, LMNS expression levels (represented by two genes that code for different isoforms (Ml_17628 and Ml_17636); Vining et al., 2022) were low in roots and high in stems (**Supplemental Table S5**; Vining and Pandelova, 2022), consistent with the volatile quantities emitted from these organs as determined in the present study. The same gene expression patterns and volatile emission characteristics were observed for *M. longifolia* CMEN 584 (**Supplemental Tables S4**, **S5**).

We recently reported on the characterization of an MTS from *M. longifolia* CMEN 585 that generates (–)-β-pinene as its main product (Kim et al., 2022), which is an intermediate in the biosynthesis of (+)-pinocarvone (**Figure 1**). Both volatiles were emitted by stems but not from roots, which correlated well with gene expression patterns (the transcript for (–)-β-pinene synthase (Ml_39080) was expressed at relatively high levels in stems but barely detectable in roots of *M. longifolia* CMEN 585 and CMEN 584; Vining and Pandelova, 2022) (**Supplemental Tables S4**, **S5**). The gene coding for (–)-bornyl diphosphate synthase (Ml_38055) was expressed at very low levels in roots and high levels in stems, which correlated with the release of borneol (high concentration in stem volatiles for both *M. longifolia* CMEN 585 and CMEN 584; Vining and Pandelova (2022); low abundance in volatile blends emitted by roots) (**Supplemental Tables S4**, **S5**). Transcript levels for γ-terpinene synthase (Ml_14850) were previously reported to be low in both roots and stems of *M. longifolia* CMEN 585 and CMEN 584 (Vining and Pandelova, 2022), which correlated with low levels of the corresponding volatiles (γ-terpinene and p-cymene) in the same samples, as reported in the current study (**Supplemental Tables S4**, **S5**). The α-terpineol synthase gene (Ml_15999) was demonstrated earlier to be expressed at high levels in roots of *M. longifolia* CMEN 585 (Vining and Pandelova, 2022), where α-terpineol is a major volatile; expression levels of this gene in stems of *M. longifolia* CMEN 585 and both roots and stems of *M. longifolia* CMEN 584 were significantly lower (Vining and Pandelova, 2022), coinciding with a lower emission of α-terpineol from the same samples (**Supplemental Tables S4**, **S5**). In summary, there was a tight correlation with MTS gene expression patterns and the volatiles generated by these enzymes across two mint accessions and multiple organs.

### Comparison of putative active site residues across functionally diverged MTSs

(+)-α-Terpineol, which is released prominently by roots of peppermint and *M. longifolia* CMEN 585 when maintained in growth chambers while being prepared for *V. dahliae* inoculation (**Figures 1**, **3**). A phylogenetic analysis placed (+)-α-terpineol synthase (ML_15999) in the same clade as two previously characterized α-terpineol synthases (ATERS1 and ATERS2 in **Figure 4**) of Cretan thyme (Lima et al., 2013). The *M. longifolia* candidate Ml_15999 is the first α-terpineol synthase for which the product stereochemistry has been established (with an enantiomeric excess of 95.4% for the (+)- over the (–)-enantiomer). Since (+)-linalool (14.4%) and (–)-linalool (13.5%) are other notable products, ML_15999 generates almost exclusively monoterpenoid alcohols (94.2%). 1,8-Cineole was detected as a volatile emitted by greenhouse-grown peppermint and *M. longifolia* CMEN 585 but also by roots of *M. longifolia* CMEN 585 maintained in growth chambers (**Figures 1**, **3**), and, in the current study, we identified and functionally characterized a 1,8-cineole synthase (Mp_12413). This enzyme clustered with previously characterized 1,8-cineole synthases of the Lamiaceae (Wise et al., 1998; Kampranis et al., 2007; Demissie et al., 2012). Interestingly, while 1,8-cineole synthases and α-terpineol synthases of the Solanaceae were demonstrated to be very closely related (Fähnrich et al., 2011), those of the Lamiaceae occupy more distant branches of the phylogenetic tree (**Figure 4**). Signature sequence motifs also differed between members of these enzyme classes: variable region 1 was I/T-I/L-L-I-T/S in α-terpineol synthases and N-A-L-V-T in 1,8-cineole synthases; the metal ion-binding DDxxD motif was D-D-V-Y-D in α-terpineol synthases and D-D/E-V/I-F/Y-D in 1,8-cineole synthases; and variable region 2 was S-I-G/A-S-L-T/P-I in α-terpineol synthases and S-I-G-G-L/V/I-P-I in 1,8-cineole synthases (**Supplemental Figure S1**).

A gene coding for a γ-terpinene synthase from *M. longifolia* CMEN 585 was identified and characterized as part of the present work (ML_14850), which is likely responsible for the accumulation of γ-terpinene as a constituent of root volatile blends emitted by *M. longifolia* CMEN 584 and could also serve to provide the precursor for *p*-cymene formation in all species investigated here (**Figures 1** and **2**). Based on a phylogenetic analysis, the *M. lognifolia* γ-terpinene synthase was closely related to previously characterized γ-terpinene synthases (GTSs) from *Thymus caespititius* and *Ocimum vulgare* (Crocoll et al., 2010; Mendes et al., 2014), positioned in a sister clade to α-terpineol synthases (**Figure 4**). Signature sequence motifs were very similar across members of these enzyme classes but there were also some notable differences: variable region 1 was I/T-I/L-L-I-T/S in α-terpineol synthases and I-T-F-V-T in γ-terpinene synthases; the metal ion-binding DDxxD motif was D-D-V-Y-D in both; and variable region 2 was S-I-G/A-S-L-T/P-I in α-terpineol synthases and S-I-S-S-P-T-I in γ-terpinene synthases. It will now be interesting to investigate the structure-function relationships underlying these different, but closely related, activities for the formation of α-terpineol, γ-terpinene, and 1,8-cineole (**Supplemental Figure S2**).

(–)-Borneol and (–)-camphor were detected in volatiles blends released by all species investigated here, and the newly discovered MTS from *M. longifolia* accession CMEN 585 catalyzing the formation of (–)-bornyl diphosphate (ML_38055) would provide the precursor for these volatiles (**Figures 1**–**3**). This enzyme clusters with characterized bornyl diphosphate synthases (BPPSs) of *Lavandula angustifolia* and *Phyla dulcis* for which the product stereochemistry had not been assessed (Despinasse et al., 2017; Hurd et al., 2017). It is interesting to note that ML_38055 is positioned on a different branch than *Salvia officinalis* (+)-bornyl diphosphate synthase (Wise et al., 1998) (**Figure 4**). Signature sequence motifs differed between members of this enzyme class that generate the (+)-isomer and those that cluster with the (–)-isomer-releasing ML_38055: conserved region 1 was I-I-V-L-A in (+)-bornyl diphosphate synthase but L-F-V/I-L/F-I in those that cluster with (–)-bornyl diphosphate synthase; the metal ion-binding DDxxD motif was D-D-I-Y/F/S-D across all bornyl diphosphate synthases; and conserved region 2 was S-V-A-S-P-A-I in but S-I-S-A/T-H/P-L/T-I in those that cluster with (–)-bornyl diphosphate synthase (**Supplemental Figure S3**). Follow-up work beyond the scope of the current study can now be undertaken to investigate how these sequence differences affect the stereochemistry of the product.

## Conclusions

The present work demonstrates that unique blends of volatiles are emitted from roots, rhizomes and stems of several mint species, organs that had not previously been investigated in this context. Above-ground material of members of the mint family is commercially distilled to extract essential oils, which are then formulated into a myriad of consumer products. Most of the research aimed at characterizing the processes involved in the formation of terpenoid oil constituents has focused on leaves. We now demonstrate, by investigating three mint species, peppermint (*Mentha* **ˣ** *piperita* L.), spearmint (*Mentha spicata* L.) and horsemint (*Mentha longifolia* (L.) Huds.; accessions CMEN 585 and CMEN 584), that other organs – namely stems, rhizomes and roots – also emit volatiles and that the terpenoid volatile composition of these organs can vary substantially from that of leaves, supporting the notion that substantial, currently underappreciated, chemical diversity exists. Differences in volatile quantities released by plants whose roots were dipped in a *V. dahliae*-spore suspension (experimental) or dipped in water (controls) were evident: in mint species that are susceptible to *Verticillium* wilt disease (peppermint and *M. longifolia* CMEN 584) roots showed increases in the emission of some volatiles, while the experimental treatment decreased the release of certain volatiles from rhizomes of species that show resistance to the disease (spearmint and *M. longifolia* CMEN 585). In the present study, we then identified and functionally characterized four MTSs whose expression was closely correlated with the emission of (+)-α-terpineol, 1,8- cineole, γ-terpinene, and (–)-bornyl diphosphate. In conjunction with previously described MTSs that catalyze the formation of (–)-β-pinene and (–)-limonene, the product profiles of the MTSs identified here can explain the generation of all major monoterpene skeletons represented in the volatiles released by different mint organs.

## Accession numbers

Nucleotide sequences of genes characterized as part of this study were deposited in GenBank and received the accession numbers: OP784758, (–)-bornyl diphosphate synthase (*M. longifolia*); OP784759, 1,8-cineole synthase (*M. piperita*); OP784760, (+)-α-terpineol synthase (*M. longifolia*); and OP784761, γ-terpinene synthase (*M. longifolia*).

## Data availability statement

The original contributions presented in the study are included in the article/**Supplementary Materials**. Further inquiries can be directed to the corresponding author.

## Author contributions

BL designed the research; AP, IL, LB, and NS performed the experiments; IP and KV provided training for the *V. dahliae* inoculation experiments; AP, BL, IL, LB, and NS analyzed the data; BL and MW served as co-mentors for LB; BL wrote the manuscript, with contributions from all authors. All authors contributed to the article and approved the submitted version.

## Supplementary material

Supplementary Figure 1 Alignment of sequences of characterized α-terpineol synthases and 1,8-cineole synthases of the Lamiaceae.

Supplementary Figure 2 Alignment of sequences of characterized α-terpineol synthases and γ-terpinene synthases of the Lamiaceae.

Supplementary Figure 3 Alignment of sequences of characterized bornyl diphosphate synthases of the Lamiaceae.

Supplementary Table 1 Accession numbers and amino acid sequences of terpene synthases included in a phylogenetic analysis presented in **Figure 4**.

Supplementary Table 2 Complete terpenoid profiles for the data sets included in **Table 1**.

Supplementary Table 3 Complete terpenoid profiles for the data sets included in Table 2>.

Supplementary Table 4 Complete terpenoid profiles for the data sets included in Table 3.
