# Supplementary material for: Biochemical basis for the formation of organ-specific volatile blends in mint
Source: Front Plant Sci. 2023 Apr 14;14:1125065. doi: 10.3389/fpls.2023.1125065 (PMC10140540; doi:10.3389/fpls.2023.1125065)
Supplement: Supplementary Figure 1 — Alignment of sequences of characterized α-terpineol synthases and 1,8-cineole synthases of the Lamiaceae. [file Image_1.pdf]

Supplemental Figure S1. Alignment of α-terpineol synthases (ATERS) and 1,8-cineole synthases (CINS) of the Lamiaceae. Ml_15999 and Mp_12413 are candidate monoterpene synthases characterized as part of the present study.

```
Ml_15999        RRSGNYEPSLWDFDYIQSLDTH-RYKEEKHLIREEELIVQVKMLLGK-EM--EAVKQLEL 56
ATERS1          RRSGNYEPSLWDFDFIQSLDNHHPYVKEEQLKREEELIVQVKILLGT-KM--EAVKQLEL 57
ATERS2          RRSGNYEPSLWDFDFIQSLDNHHPYVKEKQLKREEELIVQVKMLLGT-KM--EAVKQLEL 57
CINS_LAVAN      RRSGGYPPALWDFDTIQSLNT--EYKGERHMRREEDLIGQVREMLVHEEVD--LTPQLEL 56
CINS_LAVIN      RRSGGYPPALWDFDTIQSLNT--EYKGERHMRREEDLIGQVREMLVHEEVD--LTPQLEL 56
CINS_SALOF      RRTGGYQPTLWDFSTIQLFDS--EYKEEKHLMRAAGMIAQVNML-LQEEVD--SIQRLEL 55
CINS_THYAL      RRSGNYKPTFWNFDRIQSLNS--VYKEERYVRRAADLIRQVKML-LHEESDDDVLPQLEL 57
Mp_12413        RRSGNYEPTLWDFDRIQSLNS--VCTE-RDGRRAAVLIKEVKML-LQEEV-DGVLRQLEL 55
                **:*.* *::*:*. ** ::.       .   *    :* :*. :   :       :***

Ml_15999        IDDLKNLGLSYFFQDEIKKILSCIYNEHNF-SQNNKVRDLHFTALGFRLLRHHGFDVSQE 115
ATERS1          IDDLKNLGLSYFFRDEIKTILTSIYNNSFE-NKNNQVGDLYFTSLGFRLLRQHGFNVSQD 116
ATERS2          IDDLKNLGLSYFFRDEIKTILTSIYNNSFE-NKNNQVGDLYFTSLGFRLLRQHGFNVSQD 116
CINS_LAVAN      IDDLHKLGISCHFENEILQILKSIYLNQN------YKRDLYSTSLAFRLLRQNGFILPQE 110
CINS_LAVIN      IDDLHKLGISCHFENEILQILKSIYLNQN------YKRDLYSTSLAFRLLRQNGFILPQE 110
CINS_SALOF      IDDLRRLGISCHFDREIVEILNSKYYTNN----EIDESDLYSTALRFKLLRQYDFSVSQE 111
CINS_THYAL      IDDLRGLAISCHFDEEIERILNHLFHHD-----DVEEGDLYSTSLTFKLLRQHGFNISQG 112
Mp_12413        IDDLQRLGISCHFNEEIKQILNSFYYNEFNDAIVAEERDLYFTALAFRLLRQHGFNVSQE 115
                ****: *.:* .*  **  **.  :             **: *:* *:***: .* : *

Ml_15999        IFDCFKNEEGSDFEKALIGEDMKGILQLYEASFLLREGEDTLELARKFSTKYLQKRVDEG 175
ATERS1          IFDCFKNEKGSDFDETLIGEDTKATLQLYEASFHLREGENTLELARQISTKYLQKKVNEG 176
ATERS2          IFDCFKNEKGSDFDETLIGEDTKATLQLYEVSFHLREGENTLELARQISTKYLQKQVNEG 176
CINS_LAVAN      VFDCFKNEKGT-DFKPSLSHDRKGLLQLYEASFLSRQGEETLQLAREFATKILQKEVDE- 168
CINS_LAVIN      VFDCFKNEKGT-DFKPSLSHDRKGLLQLYEASFLSRQGEETLQLAREFATKILQKEVDE- 168
CINS_SALOF      VFDCFKNDKGT-DFKPSLVDDTRGLLQLYEASFLSAQGEETLHLARDFATKFLHKRVLVD 170
CINS_THYAL      LFEHFKCEDGT-DFKPIHVEDTKGLLQLYEASFLSTRGEETLELATQFARKSLQEKLLDH 171
Mp_12413        VFDYFKSEEGIDDFKTIHAEDTKGLLQLYEASFLSTQGEETLELAREYALKFLQKI-LDH 174
                :*: ** :.*    :    .* :. *****.**   .**:**.** . : * *::

Ml_15999        IINDDNNNNILSWIRHSLDLPLHWRIQRLEARWFLDAYSTRKDMNPLIFELSILDFNNIQ 235
ATERS1          RISDE---NLSSWIRHSLDLPLHWRIQRLEARWFLDAYAVREDKNPLIFELAKLDFNIIQ 233
ATERS2          RISDE---NLSLWIRHSLDLPLHWRIQRLEARWFLDAYAAREDKNPLIFKLAKLDFNIIQ 233
CINS_LAVAN      -----------RDFETKMGFPSHWRVQMPNARLHIDAYRKRTDMNPVVLELAILDTNIVQ 217
CINS_LAVIN      -----------RDFETKMGFPSHWRVQMPNARLHIDAYRKRTDMNPVVLELAILDTNIVQ 217
CINS_SALOF      KDI-----NLLSSIERALELPTHWRVQMPNARSFIDAYKRRPDMNPTVLELAKLDFNMVQ 225
CINS_THYAL      HEID-N-QYILSSIRDALEIPSHWRVRTPYAISFIDAYKKRPLMNPTVLELAILDINIIQ 229
Mp_12413        EIINDE-NLSSSILRDAIKIPIHWRVQMPNARSYIDAYERKPRMHPIVLELAKLEITIVQ 233
                             :.  : :* ***::   *  .:***  :   :* :::*: *: . :*
                                                           Variable Region 1
Ml_15999        ATHILELKEVSRWWNNSCLAEKLPFVRDRVVESFFWAAGLFEGHEYGYQRKMVASIIILI 295
ATERS1          ATQQEELKEVSRGWNDSCLAEKLPFVRDRVVESYFWGVGLFEGHEFGYQRKLTAANTLLI 293
ATERS2          ATQQEELKEVSRWWNDSCLAEKLPFVRDRVVESYFWGVGLFEGHEFGYQRKLTAAYILLI 293
CINS_LAVAN      AQFQEELKETSRWWESTSLVQELPFVRDRIVECYLWTTGVIQRREHGYERIMLTKINALV 277
CINS_LAVIN      AQFQEELKETSRWWESTSLVQELPFVRDRIVECYLWTTGVIQRREHGYERIMLTKINALV 277
CINS_SALOF      AQFQQELKEASRWWNSTGLVHELPFVRDRIVECYYWTTGVVERREHGYERIMLTKINALV 285
CINS_THYAL      AQFQEELKEASRWWNGTGLVQQLPFVRDRIVECYFWTTGVLERRQHVYERIMLTKINALV 289
Mp_12413        AQFQQELKETSRWWHSTSLVQQLPFVRDRIVECYYWTTGVLERREHGYERIMLTKINALV 293
                *    ****.** *..: *..:*******:**.: * .*:.: ::. *:* : :    *:
                   DDxxD Motif
Ml_15999        TAIDDVYDVYGTLGELQLFTDTIRRWDTESIDQLPYYMQLCYLALYNYVSNLAYDILKDR 355
ATERS1          SAIDDVYDVYGTLDELRLFTDVFRRWDTESIDQLPYYMQLCYLALYNYVSGVAYDILKDH 353
ATERS2          SAIDDVYDVYGTLDELRLFTDVFRRWDTESIDQLPYYMQLCYLALHNYVSGVAYDILKDH 353
CINS_LAVAN      TTIDEVFDIYGTLEELQLFTTTIQRWDLESMKQLPPYMQLCYLALHKFVIEEAYETLKEK 337
CINS_LAVIN      TTIDDVFDIYGTLEELQLFTTTIQRWDLESMKQLPPYMQLCYLALHKFVIEEAYETLKEK 337
CINS_SALOF      TTIDDVFDIYGTLEELQLFTTAIQRWDIESMKQLPPYMQICYLALFNFVNEMAYDTLRDK 345
CINS_THYAL      TTIDDIYDVYGTIGELRLFTNAVQRWDIDSINELPPYMQLCYLALYNFVNEEAYHTLKDR 349
Mp_12413        TTIDDIYDIYGTFEELQLFTNAIKRWDIESMNQLPPYMQQCYLALQNFVNEMAYNTLKQK 353
                ::**:::*:***: **:*** ..:*** :*:.:** *** ***** ::*   **. *::
```

```
                                                                  Variable Region 2
Ml_15999        RFNTIPYLHKSWLCLVETYLKEAEWYESGYTPTLEEYLSNAKISIGSLTILLQVELSLQK 415
ATERS1          RRNTIPYLQETWVELVEAYMKEAEWYKSGYTPSLEEYLTIAKISIASLTILLSVELSLPD 413
ATERS2          RRNTIPYLQETWVELVEAYMKEAEWYQSGYTPSLEEYLTIAKISIGSLPILLSVELSLPD 413
CINS_LAVAN      GFNSIPYVTKWWVNLVESYMKEATWYYNGYKPSMQEYINNAWISIGGLPILSHLFFRFTD 397
CINS_LAVIN      GFNSIPYVTKWWVNLVESYMKEATWYYNGYKPSMQEYINNAWISIGGLPILSHLFFRFTD 397
CINS_SALOF      GFNSTPYLRKAWVDLVESYLIEAKWYYMGHKPSLEEYMKNSWISIGGIPILSHLFFRLTD 405
CINS_THYAL      GFNSIPFLRKTWIDLVETYMREAEWYHNGHNPSLGEYMENAWISIGGVPILSHLFFRLTD 409
Mp_12413        GFNSIPYLHKTWVDLVEAYMREAEWYHNGHKPSLEEYMNNAWISIGGVPILSHIFFCVTD 413
                  *: *:: : *: ***:*: ** **  *:.*:: **:  : ***..: **  : : . .

Ml_15999        STLDRT--AFDLRHKILYLSALVSRLADDLGTAPSELKRGDVPNAIQCYMKD-KNCSEEE 472
ATERS1          STIDRA--TFDRRHKMFYLSATVSRLADDLGTAPSELERGDVPKAIQCYMKD-TNASEEE 470
ATERS2          STIDRA--TFDRRHKMFYLSATVSRLADDLGTAPSELERGDVPKAIQCYMKD-TNASEEE 470
CINS_LAVAN      SIE-----SMDKYRDMDRASCTILRLADDMGTSLVEVERGDVPKAIQCYMNE-TNASEEE 451
CINS_LAVIN      SIE-----SMDKYRDMDRASCTILRLADDMGTSLVEVERGDVPKAIQCYMNE-TNASEEE 451
CINS_SALOF      SIEEEDAESMHKYHDIVRASCTILRLADDMGTSLDEVERGDVPKSVQCYMNE-KNASEEE 464
CINS_THYAL      SIDDETVERMHEYHNIVRGSCTILRLADDLGTSLDEVKRGDVPKSVQCYMNE-KNASEEE 468
Mp_12413        SIDEVTVERVHEYHDIVRASCTILRLADDLGTSLDEVKRGDVPKSVECYMNDEKNASEQE 473
                *        ..  :.:   *. : *****:**:  *::*****:::*** :: .*.**:*

Ml_15999        ARAHVRGMIGEVWKEMNTAMAVSD--DDCPFTEQVVEAAANLGRAAQFIYME---GDGHG 527
ATERS1          ARGHVRFMIGETWKELNTAMAK-P--DDCPFTEQVVEATANLGRAAQFIYRE---GDGHG 524
ATERS2          AQGHVRFMIREAWKELNTAMAE-P--DDCPFTEQVVEATANIGRAAQYIYRE---GDGHG 524
CINS_LAVAN      AREYVRRLIEKEWEKMNTETMWDDDDDDFTLSKHYCEVVANLARMAQFIYQDGLDGFGMK 511
CINS_LAVIN      AREYVRRLIEKEWEKMNTETMWDDDDDDFTLSKYYCEVVANLARMAQFIYQDGLDGFGMK 511
CINS_SALOF      AREHVRSLIDQTWKMMNKEMMTS------SFSKYFVQVSANLARMAQWIYQHESDGFGMQ 518
CINS_THYAL      AREYVRSLIEETWRTMNTELMASAD---SPFSKYFVEAAANLGRMAQCVYQHESDGFGMQ 525
Mp_12413        ARAHVRSIIKNTWKTMNEEMMTSTN---SQFSKYFVEAAANLGRMSLCIYQDECDGFGMQ 530
                *: :** :* : *. :*             :::   :. **:.* :  :* .   * *

Ml_15999        HSQIHEQMRSLLFHPYI------------------------------------------- 544
ATERS1          HFQIHQHMGNLFFHPYV------------------------------------------- 541
ATERS2          HFQIRQHVRNLFFHPYV------------------------------------------- 541
CINS_LAVAN      DSKVNKLLKELLFERYE------------------------------------------- 528
CINS_LAVIN      DSKVNKLLKELLFERYE------------------------------------------- 528
CINS_SALOF      HSLVNKMLRGLLFDRYE------------------------------------------- 535
CINS_THYAL      HSRVNTMLRSLLFDPYA------------------------------------------- 542
Mp_12413        HSRVNKMLRGLLFDPCI------------------------------------------- 547
```
