# Supplementary material for: Biochemical basis for the formation of organ-specific volatile blends in mint
Source: Front Plant Sci. 2023 Apr 14;14:1125065. doi: 10.3389/fpls.2023.1125065 (PMC10140540; doi:10.3389/fpls.2023.1125065)
Supplement: Supplementary Figure 2 — Alignment of sequences of characterized α-terpineol synthases and γ-terpinene synthases of the Lamiaceae. [file Image_2.pdf]

Supplemental Figure S2. Alignment of α-terpineol synthases (ATERS) and γ-terpinene synthases (GTS) of the Lamiaceae. Ml_15999 and Ml_14850 are candidate monoterpene synthases characterized as part of the present study.

```
GTS_ORIVU       RRSGNYQASIWNNDYVQSFNTN-QYKDEKHLKKKEELIAQV-KILLNTKMEAVKQLELIE 58
Ml_14850        RRSGNYQPSLWDFDYIQSLNTH-HYKEERQLNREEELIIQVKKMLLGEKMEAVKQLELID 59
GTS4_THYCA      RRSGNYQPSLWDFNFIQSQPTH-HYEEERN--RKEDLIVHV-KTLLGTKMEAIEQLDFID 56
Ml_15999        RRSGNYEPSLWDFDYIQSLDTH-RYKEEKHLIREEELIVQV-KMLLGKEMEAVKQLELID 58
ATERS1          RRSGNYEPSLWDFDFIQSLDNHHPYVKEEQLKREEELIVQV-KILLGTKMEAVKQLELID 59
ATERS2          RRSGNYEPSLWDFDFIQSLDNHHPYVKEKQLKREEELIVQV-KMLLGTKMEAVKQLELID 59
                ******: *:*: :::**  .:  * .*.:  ::*:** :* * **. :***::**::*:

GTS_ORIVU       DLRNLGLTYYFQDEVKKILTSIYNDHKCFKNEQVGDLYFTSLGFRLLRLHGFDVSEEVFD 118
Ml_14850        DLKNLGLSYFFQDEIKKILSCIYNEHNFFQNNKVGDLHFTALGFRLLRHHGFDVSQEIFD 119
GTS4_THYCA      DLRNLGLSYFFLDEIKNILSFIYSENKCFQNNQAGDLYFTSLGFRLLRQHGFNVSQEVFN 116
Ml_15999        DLKNLGLSYFFQDEIKKILSCIYNEHNFSQNNKVRDLHFTALGFRLLRHHGFDVSQEIFD 118
ATERS1          DLKNLGLSYFFRDEIKTILTSIYNNSFENKNNQVGDLYFTSLGFRLLRQHGFNVSQDIFD 119
ATERS2          DLKNLGLSYFFRDEIKTILTSIYNNSFENKNNQVGDLYFTSLGFRLLRQHGFNVSQDIFD 119
                **:****:*:* **:*.**: **.:    :*::. **:**:******* ***:**:::*:

GTS_ORIVU       FFKNEDGSDFKA-SLGENIKDVLQLYEASFLIREGEVILEQARVFSTKHLEKKVDEGI-- 175
Ml_14850        CFKNEEGSDFEKTLIGEDTKGMLQLYEASFLLREGEDTLELARKFSTKYLQKRVDEGIRN 179
GTS4_THYCA      RFKNDDGSDFKA-SLGEDTKGILQLYEASFLLREGEDTLELARQFSTKYLQKKVDEKG-I 174
Ml_15999        CFKNEEGSDFEKALIGEDMKGILQLYEASFLLREGEDTLELARKFSTKYLQKRVDEGIIN 178
ATERS1          CFKNEKGSDFDETLIGEDTKATLQLYEASFHLREGENTLELARQISTKYLQKKVNEGRIS 179
ATERS2          CFKNEKGSDFDETLIGEDTKATLQLYEVSFHLREGENTLELARQISTKYLQKQVNEGRIS 179
                 ***:.****.   :**: *  *****.** :****  ** ** :***:*:*:*:*

GTS_ORIVU       ND--EKLLAWIRHSLALPLHWRIQRLEARWFLDAYRARKDMIPLIFELGKIDFHIIQETQ 233
Ml_14850        DD--NNLLSWIRHSLDLPLHWRIQRLEARWFLDAYSRRKDMNPLIFELAILDFNNIQATQ 237
GTS4_THYCA      DD--ENVLSWIRHSLDLPLHWRIQRLEARWFLDAYAARKDMNPLILELGKIDFNIIQATM 232
Ml_15999        DDNNNNILSWIRHSLDLPLHWRIQRLEARWFLDAYSTRKDMNPLIFELSILDFNNIQATH 238
ATERS1          DE---NLSSWIRHSLDLPLHWRIQRLEARWFLDAYAVREDKNPLIFELAKLDFNIIQATQ 236
ATERS2          DE---NLSLWIRHSLDLPLHWRIQRLEARWFLDAYAAREDKNPLIFKLAKLDFNIIQATQ 236
                ::   ::  ****** *******************  *:*  ***::*. :**: ** *
                                                  Variable Region 1
GTS_ORIVU       LEELQEVSKWWTNSNLAEKLPFVRDRIVECYFWALGLFEPHEYGYQRKMAAIIITFVTII 293
Ml_14850        LEELKEVSRWWNSSCLAEKLPFVRDRIVECYFWALGLFEPHENGYQRKKAAIIITFVTII 297
GTS4_THYCA      LDELKEVSRWWNNSCLAEKLPFVRDRIVECYFWALALFEPHQFGYQRKMAAIIITFVTVI 292
Ml_15999        ILELKEVSRWWNNSCLAEKLPFVRDRVVESFFWAAGLFEGHEYGYQRKMVASIIILITAI 298
ATERS1          QEELKEVSRGWNDSCLAEKLPFVRDRVVESYFWGVGLFEGHEFGYQRKLTAANTLLISAI 296
ATERS2          QEELKEVSRWWNDSCLAEKLPFVRDRVVESYFWGVGLFEGHEFGYQRKLTAAYILLISAI 296
                  **:***: *..* ***********:**.:**. .*** *: ***** .*    ::: *
                DDxxD Motif
GTS_ORIVU       DDVYDVYGTLDELQLFTDAIRKWDFQSISTLPYYMQVCYLALYTYASELAYDILKDQGFN 353
Ml_14850        DDVYDVYGTLDELQLFTNTIQRWDTDTINQLPYYMQVCYLALYTFVSEMAYDILKEQGFN 357
GTS4_THYCA      DDVYDVYGTLDELQLFTDTVRRXDTESISQLPYYMQVCYLSLYTYVSELAYDILKDKGFN 352
Ml_15999        DDVYDVYGTLGELQLFTDTIRRWDTESIDQLPYYMQLCYLALYNYVSNLAYDILKDRRFN 358
ATERS1          DDVYDVYGTLDELRLFTDVFRRWDTESIDQLPYYMQLCYLALYNYVSGVAYDILKDHRRN 356
ATERS2          DDVYDVYGTLDELRLFTDVFRRWDTESIDQLPYYMQLCYLALHNYVSGVAYDILKDHRRN 356
                **********.**:***:..:: * ::*. ******:***:*:.:.* :******::  *
                                                      Variable Region 2
GTS_ORIVU       SIAYLQRSWLSLVEGFFQEAKWYYAGYTPTLAEYLENAKVSISSPTIISQVYFTLPNSTE 413
Ml_14850        SIPYLQKSWVSLVEGFFKEAQWYYKGYTPTLEEYLNNAKISISSPTIISQIYFTLPNSTE 417
GTS4_THYCA      SIPYLRRSWLSLIEGFFKEAQWYYSGYTPTLEEYLNNAKVSISSPTIISQVYFTLATSTE 412
Ml_15999        TIPYLHKSWLCLVETYLKEAEWYESGYTPTLEEYLSNAKISIGSLTILLQVELSLQKSTL 418
ATERS1          TIPYLQETWVELVEAYMKEAEWYKSGYTPSLEEYLTIAKISIASLTILLSVELSLPDSTI 416
ATERS2          TIPYLQETWVELVEAYMKEAEWYQSGYTPSLEEYLTIAKISIGSLPILLSVELSLPDSTI 416
                :* **:.:*: *:* :::**:**  ****:* ***  **:**.*  *: .: ::*  **

GTS_ORIVU       RTVVENVFGYHNILYLSGMILRLADDLGTTQFELKRGDVQKAIQCYMKDNNATEKEGAEH 473
Ml_14850        KTAIESLYEYHDILCLSGMILRLADDLGTTQFELKRGDVPKAIQCYMKDRNSTEKEAQEH 477
GTS4_THYCA      KVVXESVYGYHNILYLSGTILRLADDLGTTQFELKRGDVPKAVQCYMKDKNVTEKEAQEH 472
```

```
Ml_15999        DRT--AFDLRHKILYLSALVSRLADDLGTAPSELKRGDVPNAIQCYMKDKNCSEEEARAH 476
ATERS1          DRA--TFDRRHKMFYLSATVSRLADDLGTAPSELERGDVPKAIQCYMKDTNASEEEARGH 474
ATERS2          DRA--TFDRRHKMFYLSATVSRLADDLGTAPSELERGDVPKAIQCYMKDTNASEEEAQGH 474
                  .   .   *.:: **. : ********:  **:**** :*:****** * :*:*.  *

GTS_ORIVU       VKYLLREAWKEMNTAMA--DPECPLSEDLVDAAANLGRASQFIYLEGDGHGVQHSEIHNQ 531
Ml_14850        VRFLIREAWKEMNTAMA--DSDCPFSEELVAAAANLGRAAQYIYLEGDGHGVQHSEIHKQ 535
GTS4_THYCA      VRYLIQEAWKEMNTAMA--EPDCPFSEELVTAAANLGRASQFIYLEGDGHGVQHSEIHKQ 530
Ml_15999        VRGMIGEVWKEMNTAMAVSDDDCPFTEQVVEAAANLGRAAQFIYMEGDGHG--HSQIHEQ 534
ATERS1          VRFMIGETWKELNTAMAK-PDDCPFTEQVVEATANLGRAAQFIYREGDGHG--HFQIHQH 531
ATERS2          VRFMIREAWKELNTAMAE-PDDCPFTEQVVEATANIGRAAQYIYREGDGHG--HFQIRQH 531
                *: :: *.***:*****    :**::*::* *:**:***:*:** ******  * :*:::

GTS_ORIVU       MGGLIFEPYV        541
Ml_14850        MGGLIFEPYA        545
GTS4_THYCA      MGGLLFETYA        540
Ml_15999        MRSLLFHPYI        544
ATERS1          MGNLFFHPYV        541
ATERS2          VRNLFFHPYV        541
                : .*:*. *
```
