# Supplementary material for: Biochemical basis for the formation of organ-specific volatile blends in mint
Source: Front Plant Sci. 2023 Apr 14;14:1125065. doi: 10.3389/fpls.2023.1125065 (PMC10140540; doi:10.3389/fpls.2023.1125065)
Supplement: Supplementary Figure 3 — Alignment of sequences of characterized bornyl diphosphate synthases of the Lamiaceae. [file Image_3.pdf]

Supplemental Figure S3. Alignment of bornyl diphosphate synthases (BPPS) of the Lamiaceae. Ml_38055 is a candidate monoterpene synthase characterized as part of the present study.

```
BPPS_SALOF      --------------------------RRSGNYQPALWDSNYIQSLNTPYTEERHLDRKAE 34
BPPS_PHYDU      RRSGNYEPPIWNFNYVQSSSSQYTAGRRSGNYEANMWDFDYIQSSSSQFTEDRYLERASE 60
Ml_38055        --------------------------RRSGNYKPTLWDFDRIQSLNSVYTEEKYATRASE 34
BPPSLAVAN       --------------------------RRSGMYKPTLWDFDRIQSLNSVYTEEKYSTRACD 34
                                          **** *:  :** : *** .: :**:::  * .:

BPPS_SALOF      LIVQVRILLKEKM-EPVQQLELIHDLKYLGLSDFFQDEIKEILGVIYNEHKCFH--NNEV 91
BPPS_PHYDU      LVVQVKKLLEEELTEPIQQLELIDDLQNMGVSYHFEDEIKQILKSMYDDRVKKYNSKDSK 120
Ml_38055        LVVVVKKLLEEES-SWFGQLELIDDLQRLGLSYHFEDEIRQILSSIYLDD------KYCK 87
BPPSLAVAN       LIQQVKKLLE-ES-DWFRQLQLIDDLQRLGLSYRFDDEINLILNTIYFEK------KFCE 86
                *:  *: **: :  . . **:**.**: :*:*  *:***. **  :* :        :

BPPS_SALOF      EKMDLYFTALGFRLLRQHGFNISQDVFNCFKNEKGIDFKASLAQDTKGMLQLYEASFLLR 151
BPPS_PHYDU      NVRDLYSTALEFRLSRQHGFTISQEVFDCFKNNKG-GFEASLAEDTRGLLQLYEASFMLM 179
Ml_38055        -KMDLYSTSLCFRLLRQHGFKVSQDVFNCFKNKKG-DFESSLGEDMKGLLELYEASFLLT 145
BPPSLAVAN       KEMDLYSTSLAFRLLRQHGLKVSQEVFDCFKNEEG-DFEARLGDETNGILEMCEASFLAT 145
                   *** *:* *** ****:.:**:**:****::* .*:: *.:: .*:*:: ****:

BPPS_SALOF      KGEDTLELAREFATKCLQKKLDEGGN---EIDENLLLWIRHSLDLPLHWRIQSVEARWFI 208
BPPS_PHYDU      EGEETLEQAKEFATSFLLKKLEDDTKHGILVDENLSLSVFHALELPIHWRTQRHNARWFI 239
Ml_38055        HGEETLEQARVFSTNLLQKKLDDGE----IMDEHLLNLVRHSLRLPLHWSVQRPNARWFI 201
BPPSLAVAN       EGEETLELARLFTTNILQKKLDDERNELLIMDDYLRTLIRHSLDLPLYWRVQRPSARWFI 205
                .**:*** *: *:*. * ***::       :*: *   : *:* **::*  *  .*****

                                Variable Region 1  DDxxD Motif
BPPS_SALOF      DAYARR--PDMNPLIFELAKLNFNIIQATHQQELKDLSRWWSRLCFPEKLPFVRDRLVES 266
BPPS_PHYDU      DAYEKR--SNRNSVVLELAKVDFNIVQATYQQEIKHISRWWEQTRLAEKLPFARDRLVEN 297
Ml_38055        DACAKKRLENTNPILLELAKLDFNIVQAAHQQELKHVSRWWEESKLAEKLPFARDRVVEN 261
BPPSLAVAN       EAYATR--SDMNPIMLELAKLDFNIVQATHQEELKQVSRWWKESRLAEKLPFARDRVVEN 263
                :*   :   : * :::****::***:**::*:*:*.:****..  : *****.***:**.

BPPS_SALOF      FFWAVGMFEPHQHGYQRKMAATIIVLATVIDDIYDVYGTLDELELFTDTFKRWDTESITR 326
BPPS_PHYDU      FLWTVGWLREPQYGYARIMCTKLFIFITYVDDIFDVYGTLEELQLFRDVIRRWDIEAMGQ 357
Ml_38055        YIWNVGLLFEPQYGYPRIMTTKLFILITVIDDISDVYGTLEETQLFNDTIQRWDTEGLDK 321
BPPSLAVAN       YLWNRGMLFPPQYGYPRIMNAKLFVLITVLDDIYDVYGTLEETQLFTNLITRWDVEAIGQ 323
                ::*   * :   *:** * * :.:::: * :*** ******:* :** : : *** *.: :

                                         Variable Region 2
BPPS_SALOF      LPYYMQLCYWGVHNYISDAAYDILKEHGFFCLQYLRKSVVDLVEAYFHEAKWYHSGYTPS 386
BPPS_PHYDU      LPNYMQMCFLAIDNFINEMAYDVLKEQEFVIIPHLRKMWADLCTSYCQEAEWYYNKYMPT 417
Ml_38055        LPEYMQICYLALDSFIDEAAYHVLKEQGVLIIQDLRKSWADLCAAYAKEAEWYYTGYKPT 381
BPPSLAVAN       LPEYMRICYMAIDNNINELAYEVLKQHGLLIIQDLRKFWADLCVAYGKEAEWYYTGYKPT 383
                ** **::*: .:.. *.: **.:**:: .. :  ***  .**  :* :**:**:. * *:

BPPS_SALOF      LDEYLNIAKISVASPAIISPTYFTFANASHDTAVIDSLYQYHDILCLAGIILRLPDDLGT 446
BPPS_PHYDU      MDEYINNACISISTPLILSNTYFVVTNPIE-EEVVQNFYKNPDVVRYSAMILRLADDLGT 476
Ml_38055        LEEYMEVAWISISAHTILSYVFFLISNPIE-KDAAENLRNYHNVIRCSAMVLRLADDLGT 440
BPPSLAVAN       LEEYLEVAWVSISAHLILGYMFFLTSNPIE-KEASQSLSNYHNIIRNSAMVLRLADDLGT 442
                ::**:: * :*::: *:.  :*  :*  .   . :.: :  ::: :.::*** *****

BPPS_SALOF      SYFELARGDVPKTIQCYMKETNASEEEAVEHVKFLIREAWKDMNTAIAAG-YPFPDGMVA 505
BPPS_PHYDU      SEFEAERGDVPKAIECYMNESGASREEAREHVKFMIWEAWKKINKELLSN-ASFPQFFLR 535
Ml_38055        EPFEMRRGDVPKAVECYMNDTGASMEEGREYVKFMIRETWKETNEESFKEKLPFSEIFMR 500
BPPSLAVAN       SPYEMQRGDVPKAVECYMNENGASTEEAREYVKHLLREVWKETNGERFKE-SPFTPSFMR 501
                . :*  ******:::***::..** **. *:**.:: *.**. *         *   ::

BPPS_SALOF      GAANIGRVAQFIYLHGDGFGVQHSKTYEHIAGLLFEPYA     544
BPPS_PHYDU      NAADLGRAGQFMYQHGDGFGVNPHHHKEDVSTLFFEPL-     573
Ml_38055        SAADLGRQAQYMYQHGDGHGISNREMEERILGLIFEPIV     539
BPPSLAVAN       ICADLGRMAQFMYQHGDGHGIRNLQMEDRIQSLIFEPIV     540
                 .*::** .*::* ****.*:   .  : :  *:***
```
